# BaMV‐Vectored Compact AsCas12f1‐HKRA Enables Transgene‐Free Genome Editing in Moso Bamboo ( *Phyllostachys edulis* )

**DOI:** 10.1111/pbi.70474

**Published:** 2025-12-02

**Authors:** Lin Wu, Yuying Gu, Hongjue Guo, Jun Zhang, Jun Yang, Mengying Zhang, Huihui Wang, Liangzhen Zhao, Hangxiao Zhang, Lianfeng Gu

**Affiliations:** ^1^ College of Forestry, Basic Forestry and Proteomics Research Center, Fujian Provincial Key Laboratory of Haixia Applied Plant Systems Biology Fujian Agriculture and Forestry University Fuzhou China

**Keywords:** BaMV, *Phyllostachys edulis*, virus‐induced genome editing

Plant positive‐strand RNA viruses (PSVs) are excellent vectors for delivering gRNAs into Cas9‐overexpressing plants to achieve efficient genome editing (Shen et al. 2024; Steinberger and Voytas 2025; Wu et al. 2024). The development of a more compact programmable Cas12 system will bring revolutionary progress to plant genome editing technology, particularly in the field of plant PSV‐mediated genome editing technology (Ye et al. 2024). Bamboo mosaic virus (BaMV), the most well‐characterised bamboo PSV, has been engineered to deliver CRISPR‐Cas components, enabling DNA‐free targeted genome editing (Wu et al. 2025). Here, we optimised the dual‐promoter‐driven BaMV‐gRNA‐Cas9/Cas12f1 system with more compact and coordinated single transcript unit (STU) expression architectures, compared to compact Cas12 proteins, and validated efficacy in bamboo—establishing a versatile editing platform for woody monocots (Appendix S1).

First, we constructed two BaMV‐Cas9^*STU*^ systems (BaMV‐STU‐Cas9‐tRNA and BaMV‐STU‐Cas9) to target the endogenous *N. benthamiana Phytoene Desaturase* (*PDS*) genes (Figure 1a, Appendix S2). Following 4 weeks of agroinfiltration, molecular analysis of systemic leaves exhibiting chlorosis symptoms (Figure S1a) revealed that both BaMV‐Cas9^*STU*^ systems yielded higher levels of Cas9 transcripts and proteins compared to the BaMV‐gRNA‐Cas9 system (Figure S1b). The BaMV‐STU‐Cas9 and BaMV‐STU‐Cas9‐tRNA systems exhibited higher editing efficiencies (~58.9% and ~ 64.1%, respectively) than the BaMV‐gRNA‐Cas9 system (~48.0%), representing 1.2‐fold and 1.3‐fold increases (Figure 1b, Figures S1c, S1d). The more compact BaMV‐STU‐Cas9‐tRNA system outperforms the dual‐promoter‐driven BaMV‐gRNA‐Cas9 system (Figure S1b,e).

**FIGURE 1:**
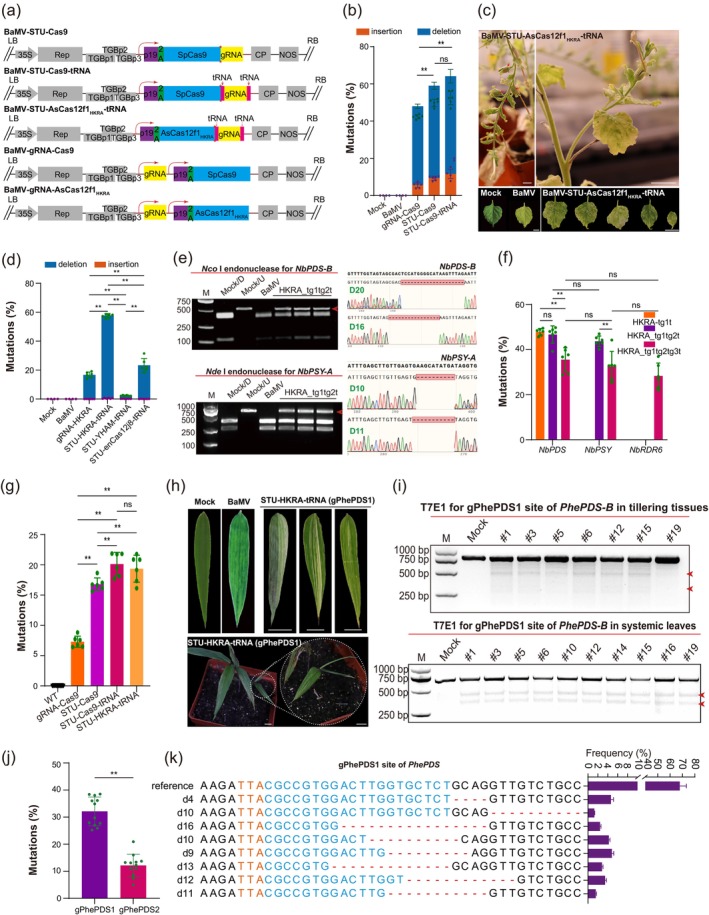
BaMV‐mediated single transcript CRISPR‐Cas system in genome editing (Details provided in Appendix S3).

Then, we engineered the BaMV‐Cas12^*STU*^ system by integrating engineered Cas12 protein variants with optimised gRNAs (Figure 1a, Figure S2a, Appendix S2). After 4 weeks of agroinfiltration, BaMV‐STU‐AsCas12f1_HKRA_‐tRNA caused distinctive white speckling, which subsequently progressed to the stems and floral sepals with increasing severity as the plants matured (Figure 1c, Figure S2b). Western blot and RT‐PCR detection showed that the BaMV‐Cas12^*STU*^ system can stably express Cas12 variants (Figure S2c). Mutation analysis revealed that BaMV‐STU‐AsCas12f1_HKRA_‐tRNA had the highest editing efficiency (57.8%), a 3.4‐fold increase over BaMV‐gRNA‐AsCas12f_HKRA_ (16.7%). It was followed by BaMV‐STU‐enCas12j8‐tRNA (23.3%) and AsCas12f1_YHAM_‐tRNA (2.5%), with deletions as the predominant edits. In contrast, the nCas12j2 and vCas12j2 systems showed no detectable activity (Figure 1d, Figures S2d, S2e). Meanwhile, no albino seeds were observed in tobacco treated with the high‐performing BaMV‐STU‐AsCas12f1_HKRA_‐tRNA system, indicating its mutagenic effects occur primarily in somatic tissues (Figure S2f).

To investigate multiplex editing, we constructed BaMV‐HKRA_tg1tg2t and BaMV‐HKRA_tg1tg2tg3t vectors targeting *N. benthamiana PDS*, *phytoene synthase* (*PSY*) and *RNA‐dependent RNA polymerase 6* (*RDR6*) genes (Figure S3a, Appendix S2). Mutation analysis confirmed effective editing at all loci (Figure 1e,f, S3b). BaMV‐HKRA_tg1tg2t achieved 56.7% and 53.7% efficiency at *NbPDS* and *NbPSY*, comparable to single‐targeting, while BaMV‐HKRA_tg1tg2tg3t only achieved 35.5% (*NbPDS*), 32.5% (*NbPSY*), and 28.3% (*NbRDR6*). Long fragment deletions (more than 13 bp) were observed (Figure S3c), demonstrating robust multiplex editing by the BaMV‐STU‐AsCas12f1_HKRA_‐tRNA system.

We previously revealed that inserting exogenous genes after BaMV ORF5 stop codon (BaMV‐EGFP‐3) enhances expression (Jin et al. 2023). Here, we constructed five vectors (HKRA_eGFP, HKRA_eGFP‐CP1/2, HKRA‐CP1/2) to compare insertion site effects (Figure S4a, Appendix S2). HKRA_eGFP‐CP1 and HKRA_eGFP‐CP2 showed GFP fluorescence only in infiltrated leaves (Figure S4b). Similarly, HKRA‐CP1 and HKRA‐CP2 also exhibited no systemic symptoms (Figure S4c). Western blot analysis on systemic leaves, inoculated leaves, and stems confirmed that relocating AsCas12f1_HKRA__tgtRNA downstream of BaMV ORF5 reduced the stable expression of AsCas12f1_HKRA_ (Figure S4d). Mutation analysis showed *NbPDS* editing efficiencies of 23.9% (HKRA‐CP1) and 55.8% (HKRA‐CP2), limited to inoculated leaves (Figures S4e, S4f). These results reveal that optimal editing and systemic spread require inserting AsCas12f1_HKRA__tgtRNA at the ORF4 stop codon combined with the STU strategy.

*Phyllostachys edulis* , the pioneer sequenced bamboo species, despite the achievement of its genetic transformation, still lags significantly behind model plants in terms of genetic foundations because of systemic bottlenecks (Huang et al. 2022; Wang et al. 2025), highlighting the imperative for novel tool development. First, we evaluated the BaMV‐gRNA‐Cas9, BaMV‐STU‐Cas9, BaMV‐STU‐Cas9‐tRNA and BaMV‐STU‐AsCas12f1_HKRA_‐tRNA systems in *P. edulis* by targeting *PheRDR6* (Figure S5a, Appendix S2). Compared with the BaMV‐gRNA‐Cas9 system (7.3%), the STU‐based systems significantly improved editing efficiency: 16.8% for BaMV‐STU‐Cas9, 20.1% for BaMV‐STU‐Cas9‐tRNA, and 19.3% for BaMV‐STU‐AsCas12f1_HKRA_‐tRNA, corresponding to 2.3‐, 2.7‐ and 2.6‐fold increases, respectively (Figure 1g, S5b). Then, we further targeted *PhePDS* loci by two different sites (gPhePDS1/2) using the more compact BaMV‐STU‐AsCas12f1_HKRA_‐tRNA system (Figure S5a, Appendix S2). After 30 days of mechanical inoculation, photobleached spots appeared in both newly emerged leaves and fresh tiller buds of gPhePDS1*‐*targeted *P. edulis* (Figure 1h, S5c). The T7EI enzyme digestion showed that the BaMV‐STU‐AsCas12f1_HKRA_‐tRNA system generated targeted editing of the *PhePDS* in both newly emerged leaves and tillering tissues (Figure 1i, Figure S5d). Deep sequencing further confirmed efficient editing, showing higher efficiency at gPhePDS1 (32.1%) than gPhePDS2 (12.1%) (Figure 1j). Deletion pattern analysis revealed long‐fragment edits at paralogous *PhePDS* loci (Figure 1k, Figure S5e). Together, these results establish the BaMV‐STU‐AsCas12f1_HKRA_‐tRNA system as an effective gene editing tool for bamboo.

In summary, we optimised the BaMV‐mediated CRISPR‐Cas genome editing system. The BaMV‐STU‐AsCas12f1_HKRA_‐tRNA system showed robust capability by enabling efficient delivery of AsCas12f1_HKRA_ and gRNA in *P. edulis* and inducing targeted mutations in newly developed tissues, highlighting its potential for accelerating bamboo breeding. Future efforts should focus on incorporating key mobile elements, such as Flowering Locus T (FT) and tRNA‐like sequences (TLS) (Ellison et al. 2020), to achieve precise gene editing in bamboo germline cells. Moreover, the design strategy of BaMV‐STU‐AsCas12f1_HKRA_‐tRNA provides a conceptual framework for developing more advanced gene editing tools based on positive stranded RNA viruses.

## Author Contributions

L.G. designed and supervised the work. L.W., Y.G. and H.G. performed the research. L.W., J.Z. and H.Z. analysed the data. L.W., J.Y., M.Z., H.W. and L.Z. prepared the sample. L.W. and L.G. wrote the paper.

## Supporting information


**Appendix S1:** pbi70474‐sup‐0001‐AppendixS1.docx. **Materials and methods**.

**Appendix S2:** pbi70474‐sup‐0002‐AppendixS2.docx. **Supplemental Tables 1‐3**.

**Appendix S3:** Figures S1–S5 (details provided in Appendix S3).
**Figure S1:** BaMV‐mediated single transcript CRISPR‐Cas9 system.
**Figure S2:** BaMV‐mediated single transcript CRISPR‐Cas12 system.
**Figure S3:** Multiplexed endogenous genome editing.
**Figure S4:** Evaluation of AsCas12f1HKRA‐tgtRNA insertions at distinct sites.
**Figure S5:** Genome editing in Phyllostachys edulis.

## Data Availability

The data that support the findings of this study are openly available in Hi‐TOM Raw data at https://doi.org/10.6084/m9.figshare.30371077.v1, reference number PBI‐01036‐2025.
