# Supplementary material for: BaMV‐Vectored Compact AsCas12f1‐HKRA Enables Transgene‐Free Genome Editing in Moso Bamboo ( Phyllostachys edulis )
Source: Plant Biotechnol J. 2025 Dec 2;24(4):2220–2. doi: 10.1111/pbi.70474 (PMC13140604; doi:10.1111/pbi.70474)
Supplement: Supplementary file 1 — Appendix S1: pbi70474‐sup‐0001‐AppendixS1.docx. Materials and methods. [file PBI-24-2220-s001.docx]

**Introduction**

The emergence of programmable CRISPR-Cas systems, with their precise and targeted genome editing capabilities, has not only significantly accelerated the widespread application of gene editing technologies in life sciences but also provided an unprecedented powerful tool for plant genetic improvement (Gong et al., 2021; Liu et al., 2022a; Zhan et al., 2021). Currently, the successful delivery of editing reagents into plant cells to generate editing events, along with subsequent plant transformation and regeneration, remains the primary bottleneck in obtaining gene-edited plants (Atkins and Voytas, 2020; Chen et al., 2019; Chen et al., 2022). To overcome these barriers, previous studies have developed various delivery approaches through persistent efforts, including transfection of DNA, RNA, and ribonucleoproteins (RNPs) into protoplasts, *Agrobacterium*-induced transfer DNA (T-DNA) transformation, particle bombardment, and virus-mediated gene editing (Chen et al., 2019; Tuncel et al., 2025). Notably, given that plant RNA viruses do not integrate into the plant genome, the utilization of plant RNA virus-mediated delivery of CRISPR-Cas system components presents a particularly attractive approach for generating non-transgenic mutant plants (Wu et al., 2024). Among these, the positive-strand RNA viruses (PSVs) have been more extensively utilized due to their relative ease in establishing reverse genetic systems compared to negative-strand RNA viruses (NSVs) (Jackson and Li, 2016). However, the PSVs have fundamental limitations, including inherent genetic instability in the expression of long exogenous genes (Shen et al., 2024) and the irregularity in the transcription start sites of viral subgenomic promoters (Oh et al., 2021). These factors pose significant technical barriers for the comprehensive delivery of CRISPR-Cas9 components by PSVs.

The utilization of ribozyme (RZ), Cys4, or tRNA elements to construct a more simplified, compact, and coordinated single transcript unit (STU) expression system comprising Cas and gRNA represents a strategy that significantly enhances editing efficiency and cross-species adaptability in plants, especially the STU-Cas9-tRNA system, which has been proven to be an effective and high-capacity platform for plant genome editing (Ellison et al., 2020; Tang et al., 2019; Tang et al., 2016; Uranga et al., 2021). Previous research has indicated that gRNA, when fused directly with the Cas9 coding sequence, can be processed by unknown ribonucleases to produce a functional CRISPR-Cas9 ribonucleoprotein complex, enabling efficient target gene editing (Mikami et al., 2017). This strategy has also been applied to *Potato Virus X* (PVX) to achieve effective gene editing in plant inoculated leaves (Ariga et al., 2020; Lee et al., 2024). These strategies provide the possibility of utilizing the PSV vectors to simultaneously deliver all components of CRISPR-Cas.

The Cas12 nucleases evolved from TnpB are classified as Class II and Class V effectors and can be further categorized into 14 distinct subtypes (Cas12a to Cas12n), with protein sizes ranging from 400 to 1400 amino acids (Koonin et al., 2023; Tang and Ji, 2024). Each subtype of Cas12 nucleases displays different structural and functional characteristics, highlighting the remarkable functional diversity (Chen et al., 2023; Lin et al., 2025). The development of an ultra-compact and programmable Cas12 nuclease system will bring revolutionary progress to plant genome editing technology, particularly in the field of plant virus mediated genome editing technology (Lin et al., 2025; Tang and Ji, 2024). Recently, several miniature Cas12 proteins, including *Acidibacillus sulfuroxidans* Cas12f1 (AsCas12f1, 422 amino acids) (Wu et al., 2021) and Cas12j from huge phages (700-800 amino acids) (Pausch et al., 2020), have garnered attention for their compact size, approximately one-third to one-half the size of SpCas9 (1368 amino acids), and their distinct protospacer adjacent motif (PAM) preferences (5′-TTR-3′ [R = A/G] or 5′-TBN-3′ [B = G/T/C], respectively). These engineered systems have demonstrated effective genome editing capabilities in plants (Bai et al., 2025; Ishibashi et al., 2024; Li et al., 2023; Liu et al., 2022b; Sun et al., 2024b; Ye et al., 2024), providing enormous potential for further advancing the use of RNA viruses, especially PSVs, to deliver Cas nucleases for achieve DNA-free genome editing in plants.

*Bamboo mosaic virus* (BaMV), belonging to the *Potexvirus* genus of *Flexiviridae*, is the most extensively studied virus infecting bamboo plants, has a flexuous morphology with a single-stranded positive-sense RNA genome (Hsu et al., 2018). The RNA genome of BaMV includes a 5' m^7^GpppG (cap0) structure and a 3' poly (A) tail and can be functionally divided into a 5' untranslated region (UTR), five ORFs, and a 3' UTR (Meng and Lee, 2017). Among them, ORF1 is involved in viral replication (Na-Sheng Lin, 1994), and ORF2, 3 and 4 are partially overlapping triple gene blocks (TGBs), which synthesize proteins TGBp1, TGBp2 and TGBp3, respectively, and are involved in the movement of viruses between host cells (Lin et al., 2004). ORF5 expresses the BaMV coat protein (CP), which is the only structural protein required to assemble BaMV virions and is responsible for the intercellular and long-distance movement of BaMV in the host (Meng and Lee, 2017; Na-Sheng Lin, 1994). BaMV exhibits tremendous potential as a versatile viral vector with diverse applications due to its exceptional cargo capacity. For instance, it can be engineered as a vaccine platform to express antigens of foot-and-mouth disease virus (FMDV) and infectious bursal disease virus (IBDV) (Chen et al., 2012; Yang et al., 2007). Additionally, BaMV efficiently expresses both exogenous and endogenous genes in bamboo (Jin et al., 2023), further highlighting its utility in plant biotechnology. Previously, we developed the BAMV-mediated CRISPR-Cas9 gene editing system (BaMV-gRNA-Cas9 system) and successfully achieved DNA-free target genome editing in *Nicotiana benthamiana* and bamboo (Wu et al., 2025). However, the earlier version of the BaMV-Cas9 system still has significant limitations due to its relatively low efficiency.

In this study, we first further optimized the previously developed BaMV-gRNA-Cas9 system by applying the STU strategy and constructed two optimized systems: the BaMV-STU-Cas9 system and the BaMV-STU-Cas9-tRNA system. We established that the STU strategy markedly improved the editing efficiency of the BaMV-mediated CRISPR-Cas9 system, with the BaMV-STU-Cas9-tRNA system demonstrating superior performance. Subsequently, we further developed the BaMV-Cas12*^STU^* system utilizing miniature Cas12 family members (AsCas12f1, Cas12j2, and Cas12j8) for plant genome editing applications. We found that the BaMV-STU-AsCas12f1_HKRA_-tRNA system, engineered using the AsCas12f1_HKRA_ variant, achieved high editing efficiency. Furthermore, our study demonstrated that the BaMV-STU-AsCas12f1_HKRA_-tRNA system enables efficient multiplex genome editing, with editing efficiencies comparable to single-gene targeting. Most significantly, this study provides definitive evidence that the BaMV-STU-AsCas12f1_HKRA_-tRNA system can achieve efficient gene editing in *Phyllostachys edulis*. In summary, the BaMV-STU-AsCas12f1_HKRA_-tRNA system developed in this study using BaMV vector combined with STU expression strategy provides a valuable approach for gene function analysis and represents a powerful tool for future molecular breeding research in bamboo species.

**Methods**

**Plant materials and growth conditions**

*Nicotiana benthamiana* plants cultured at 28°C in a greenhouse with a 16 h light/8 h dark photoperiod. Peeled *Phyllostachys edulis* seeds were soaked in water for 24 h, then evenly sown on nutrient-enriched soil and covered with 1-cm thick nutrient soil. The sown seeds were transferred to a glasshouse at 23–25°C under a 16 h light/8 h dark photoperiod for germination and subsequent cultivation.

**Construction of BaMV-based plant genome editing vectors**

The vectors used in this study were constructed based on pCAMBIA1302-BaMV-Cas9 backbone (Jin et al., 2023; Wu et al., 2025). The DNA sequences for the vCas12j2 and nCas12j2 genes were codon-optimized for *Oryza sativa* and synthesized by Tsingke Biotechnology. The enCas12j8 gene was cloned from the *Ubi:en4Cas12j8-crRNA-Rz* (Bai et al., 2025). The AsCas12f1_HKRA_ and AsCas12f1_YHAM_ genes were cloned from the recombinant vector (Ye et al., 2024). The plasmids were constructed using standard molecular cloning techniques, including PCR amplification, restriction enzyme digestion, and ligation. Amplification of all constructions was performed using high-fidelity enzyme 2x Phanta Flash Master Mix (Dye Plus) (P520, Vazyme). Following digestion with FastDigest *Xba* I (FD0684, Thermo Scientific), the PCR products were ligated using Hieff Clone Universal II One Step Cloning Kit (10923ES20, Yeasen). Ligated products were transformed into DH5𝛼 E. coli cells. All clones were confirmed by Sanger sequencing. All targeted sequences in our study are listed in Table S1. The sequences of Cas proteins expression cassettes and their corresponding gRNA used in this study are shown in Table S2. All primers used for vector construction are listed in Table S3.

In BaMV-STU-Cas9-tRNA and BaMV-STU-Cas9 vectors, the gRNAs or tRNA^Gly^-gRNA-tRNA^Gly^ elements were directly fused to the SpCas9 stop codon, processed into mature gRNAs through plants endogenous cellular mechanisms, and subsequently assembling with SpCas9 protein into functional ribonucleoprotein complex (Figure 1a, Figure S1a). To construct the BaMV-mediated BaMV-STU-Cas12-tRNA genome editing system, we used employing engineered variants — AsCas12f1 (including AsCas12f1_HKRA_ [I123H/D195K/D208R/V232A] and AsCas12f1_YHAM_ [F48Y/S188H/V232A/E316M]), Cas12j2 (including nCas12j2 [E159A/S160A/S164A/D167A/E168A] and vCas12j2 [with GSSG replacing Δ155-176]), and enhanced enCas12j8 (A121Q/S196Q) — along with their corresponding optimized gRNAs to replace the SpCas9-tgtRNA components (Figure S2a). Based on the BaMV-STU-AsCas12f1_HKRA_ system, multiple gRNAs were linked using tRNA^Gly^ to form either tRNA^Gly^-gRNA1-tRNA^Gly^-gRNA2-tRNA^Gly^ (tg1tg2t) or tRNA^Gly^-gRNA1-tRNA^Gly^-gRNA2-tRNA^Gly^-gRNA3-tRNA^Gly^ (tg1tg2tg3t) structures (Figure S3), to evaluate the multiplex gene-editing capability of the BaMV-STU-AsCas12f1HNRA system. To generate the BaMV-STU-AsCas12f1_HKRA_-tRNA-CP1 (HKRA-CP1) vector, we fused AsCas12f1_HKRA__tgtRNA directly to BaMV ORF5 by 2A peptide linker after removing stop codon, and BaMV-STU-AsCas12f1_HKRA_-tRNA-CP2 (HKRA-CP2) vector was constructed by inserting the AsCas12f1_HKRA__tgtRNA element at ORF5 termination site, with driven by ORF5 subgenomic promoter (Figure S4a). The pBaMV-2-AsCas12f1HKRA-eGFP (HKRA_eGFP), pBaMV-3-CP-2A-AsCas12f1HKRA-eGFP (HKRA_eGFP-CP1), and pBaMV-3-CP-ORF5promoter-AsCas12f1HKRA-eGFP (HKRA_eGFP-CP2) vectors were constructed at the same insertion sites (Figure S4a).

**Plant inoculation procedures**

All constructed BaMV-based plasmids were transferred into *Agrobacterium tumefaciens* strain GV3101 by freeze-thaw method for subsequent tobacco leaf infiltration. The *Agrobacterium* was cultured on Yeast Extract Mannitol Broth (YEB) medium at 28°C and shaking at 200 rpm until an OD_600_ 0.5–0.8 was reached. Subsequently, the *Agrobacterium* was washed with tobacco infection buffer (10 mM MES, 10 mM MgCl_2_, 100 μM acetylsyringane, pH 5.6). Finally, the *Agrobacterium* was resuspended in the tobacco infection buffer and incubated at 25°C in darkness for 2h before tobacco leaves inoculating. For mechanical inoculation, the inoculated tobacco leaves after 10 days of *Agrobacterium*-induced infiltration were ground with virus infection buffer (50 mM K_3_PO_4_, 1% PVP, 1‰ 2-Hydroxy-1-ethanethiol, pH 8.0) and diamond sand. Then, the juice obtained from grinding was immediately used for the mechanical inoculation of *P. edulis* leaves, with inoculated seedlings maintained under standard glasshouse conditions (23–25°C).

**RNA extraction and reverse transcription PCR (RT-PCR) detection**

Total RNA was extracted from 0.1g fresh tissue of *N. benthamiana* and *P. edulis* using FastPure Universal Plant Total RNA Isolation Kit (RC411-01, Vazyme), with spectrophotometer (DS-11 FX+, DeNovix) for quantitative assessment. According to the protocol of NovoScript Plus All-in-one 1st Strand cDNA Synthesis SuperMix (gDNA Purge) (E047, Novoprotein), first-stranded cDNA was synthesized from 2 μg total RNA, with addition of BaMV 3’-UTR specific primers (Ba32). All RT-PCR primer sequences are listed in Table S3.

**DNA extraction and PCR-restriction digestion (PCR-RE) detection**

Gnomic DNA was extracted from 0.2g *N. benthamiana* and *P. edulis* fresh tissue following FastPure Plant DNA Isolation Mini Kit (DC104-01, Vazyme) protocol. PCR amplification of gRNA target genes was performed using 2x Phanta Flash Master Mix (Dye Plus) (P520, Vazyme). Purified PCR products were subjected to restrictive digestion using FastDigest *Nco* I (FD0574, Thermo Scientific), FastDigest *Nde* I (FD0583, Thermo Scientific), FastDigest *Pvu* II (FD0634, Thermo Scientific) or T7 Endonuclease I (EN303, Vazyme). Digested fragments were then detected via electrophoresis on 2% agarose gels. All primer sequences are listed in Table S3.

**Protein extraction and immunoblotting**

For protein extraction, 0.2g fresh tissue samples form *N. benthamiana* and *P. edulis* were quickly frozen in liquid nitrogen and ground into powder. Subsequently, the powder sample was homogenized in 200 μL SDS-PAGE protein loading buffer (25 mM Tris–HCl pH 6.8, 0.5 M DTT, 10% (w/v) SDS, 0.5% (w/v) bromophenol blue, and 50% (v/v) glycerol), followed by heating at 100°C for 10 min. Proteins were separated using 10 % SDS-polyacrylamide gel electrophoresis subsequently transferred onto 0.45 μm nitrocellulose (NC) membrane (PN0454, LABLEAD). Loading consistency was confirmed by staining the NC membrane with Ponceau S. Then the membrane was blocked with TBST (20 mM Tris-base, 150 mM NaCl and 0.05% (v/v) Tween 20 pH 7.5) containing 5% (w/v) skim milk powder for 2h at room temperature. The closed NC membrane was incubated with the primary antibody in TBST for 2h at room temperature. After three TBST washes (10 min each), the membrane was incubated with the secondary antibodies containing TBST for 1.5 h at room temperature. Finally, after three TBST washes (10 min each), the membrane was incubated with chemiluminescent substrate in the dark for 2 min and subsequently analyzed using the Amersham Imager 600.

**Sanger sequencing and amplicon deep sequencing**

For Sanger sequencing, 200 ng of PCR products were digested with corresponding restriction enzymes, followed by recovery of the digestion-resistant bands, which were then TOPO-cloning into the easy vector using the Universal Zero TOPO TA/Blunt Cloning Kit (10906ES20, Yeasen). For amplicon deep sequencing, we designed Hi-TOM sequencing primers for the target genes according to the Hi-TOM Rapid Sequencing Service Protocol (<http://www.hi-tom.net/hi-tom/>) (Sun et al., 2024a). PCR amplicons were sequenced, and the clean sequences were aligned to reference sequences using the Hi-Tom online platform and SuperDecode software (Li et al., 2025). Editing efficiencies were calculated as the percentage of mutated reads relative to total mapped reads.

**Statistical analysis**

Ordinary ANOVA tests were performed with GRAPHPAD PRISM v.10.2.0 (GraphPad Software) to compare editing efficiencies across different groups. Data are presented as mean values ± standard deviation (SD) unless otherwise specified. All experiments were repeated at least three times independently.

**Data availability statement**

The raw data of genome editing analysis generated in this study have been deposited in Figshare (https://figshare.com/) under the URL: https://doi.org/10.6084/m9.figshare.30371077.v1.

**References**

Ariga, H., Toki, S. and Ishibashi, K. (2020) Potato Virus X Vector-Mediated DNA-Free Genome Editing in Plants. *Plant Cell Physiol* **61**, 1946-1953.

Atkins, P.A. and Voytas, D.F. (2020) Overcoming bottlenecks in plant gene editing. *Curr Opin Plant Biol* **54**, 79-84.

Bai, S., Cao, X., Hu, L., Hu, D., Li, D. and Sun, Y. (2025) Engineering an optimized hypercompact CRISPR/Cas12j‐8 system for efficient genome editing in plants. *Plant biotechnology journal*.

Chen, K., Wang, Y., Zhang, R., Zhang, H. and Gao, C. (2019) CRISPR/Cas Genome Editing and Precision Plant Breeding in Agriculture. *Annu Rev Plant Biol* **70**, 667-697.

Chen, T.H., Chen, T.H., Hu, C.C., Liao, J.T., Lee, C.W., Liao, J.W., Lin, M.Y., Liu, H.J., Wang, M.Y., Lin, N.S. and Hsu, Y.H. (2012) Induction of protective immunity in chickens immunized with plant-made chimeric Bamboo mosaic virus particles expressing very virulent Infectious bursal disease virus antigen. *Virus Res* **166**, 109-115.

Chen, W., Ma, J., Wu, Z., Wang, Z., Zhang, H., Fu, W., Pan, D., Shi, J. and Ji, Q. (2023) Cas12n nucleases, early evolutionary intermediates of type V CRISPR, comprise a distinct family of miniature genome editors. *Molecular Cell* **83**, 2768-2780.e2766.

Chen, Z., Debernardi, J.M., Dubcovsky, J. and Gallavotti, A. (2022) Recent advances in crop transformation technologies. *Nat Plants* **8**, 1343-1351.

Ellison, E.E., Nagalakshmi, U., Gamo, M.E., Huang, P.J., Dinesh-Kumar, S. and Voytas, D.F. (2020) Multiplexed heritable gene editing using RNA viruses and mobile single guide RNAs. *Nat Plants* **6**, 620-624.

Gong, Z., Cheng, M. and Botella, J.R. (2021) Non-GM Genome Editing Approaches in Crops. *Front Genome Ed* **3**, 817279.

Hsu, Y.-H., Tsai, C.-H. and Lin, N.-S. (2018) Editorial: Molecular Biology of Bamboo mosaic Virus—A Type Member of the Potexvirus Genus. *Frontiers in microbiology* **9**.

Ishibashi, K., Sukegawa, S., Endo, M., Hara, N., Nureki, O., Saika, H. and Toki, S. (2024) Systemic delivery of engineered compact AsCas12f by a positive-strand RNA virus vector enables highly efficient targeted mutagenesis in plants. *Frontiers in plant science* **15**.

Jackson, A.O. and Li, Z. (2016) Developments in Plant Negative-Strand RNA Virus Reverse Genetics. *Annual review of phytopathology* **54**, 469-498.

Jin, Y., Wang, B., Bao, M., Li, Y., Xiao, S., Wang, Y., Zhang, J., Zhao, L., Zhang, H., Hsu, Y.H., Li, M. and Gu, L. (2023) Development of an efficient expression system with large cargo capacity for interrogation of gene function in bamboo based on bamboo mosaic virus. *J Integr Plant Biol*.

Koonin, E.V., Gootenberg, J.S. and Abudayyeh, O.O. (2023) Discovery of Diverse CRISPR-Cas Systems and Expansion of the Genome Engineering Toolbox. *Biochemistry* **62**, 3465-3487.

Lee, S.-Y., Kang, B., Venkatesh, J., Lee, J.-H., Lee, S., Kim, J.-M., Back, S., Kwon, J.-K. and Kang, B.-C. (2024) Development of virus-induced genome editing methods in Solanaceous crops. *Horticulture Research* **11**.

Li, F., Tan, X., Li, S., Chen, S., Liu, L., Huang, J., Li, G., Lu, Z., Wu, J., Zeng, D., Luo, Y., Dong, X., Ma, X., Zhu, Q., Chen, L., Liu, Y.G., Chen, C. and Xie, X. (2025) SuperDecode: An integrated toolkit for analyzing mutations induced by genome editing. *Mol Plant* **18**, 690-702.

Li, Z., Zhong, Z., Wu, Z., Pausch, P., Al-Shayeb, B., Amerasekera, J., Doudna, J.A. and Jacobsen, S.E. (2023) Genome editing in plants using the compact editor CasPhi. *Proceedings of the National Academy of Sciences of the United States of America* **120**, e2216822120.

Lin, M.-K., Chang, B.-Y., Liao, J.-T., Lin, N.-S. and Hsu, Y.-H. (2004) Arg-16 and Arg-21 in the N-terminal region of the triple-gene-block protein 1 of Bamboo mosaic virus are essential for virus movement. *Journal of General Virology* **85**, 251-259.

Lin, W., Li, C., Li, M. and Guan, Y. (2025) Emerging nucleases in crop genome editing: towards intellectual property independence and technical flexibility. *Seed Biology* **4**, 0-0.

Liu, G., Lin, Q., Jin, S. and Gao, C. (2022a) The CRISPR-Cas toolbox and gene editing technologies. *Molecular Cell* **82**, 333-347.

Liu, S., Sretenovic, S., Fan, T., Cheng, Y., Li, G., Qi, A., Tang, X., Xu, Y., Guo, W., Zhong, Z., He, Y., Liang, Y., Han, Q., Zheng, X., Gu, X., Qi, Y. and Zhang, Y. (2022b) Hypercompact CRISPR-Cas12j2 (CasPhi) enables genome editing, gene activation, and epigenome editing in plants. *Plant Commun* **3**, 100453.

Meng, M. and Lee, C.-C. (2017) Function and Structural Organization of the Replication Protein of Bamboo mosaic virus. *Frontiers in microbiology* **8**.

Mikami, M., Toki, S. and Endo, M. (2017) In Planta Processing of the SpCas9-gRNA Complex. *Plant Cell Physiol* **58**, 1857-1867.

Na-Sheng Lin, B.-Y.L., Neng-Wen Lo, Chung-Chi Hu, Teh-Yuan Chow, Yau-Heiu Hsu (1994) Nucleotide sequence of the genomic RNA of bamboo mosaic potexvirus. *The Journal of general virology* **75**, 2513-2518.

Oh, Y., Kim, H., Lee, H.J. and Kim, S.G. (2021) Ribozyme-processed guide RNA enhances virus-mediated plant genome editing. *Biotechnol J*, e2100189.

Pausch, P., Al-Shayeb, B., Bisom-Rapp, E., Tsuchida, C.A., Li, Z., Cress, B.F., Knott, G.J., Jacobsen, S.E., Banfield, J.F. and Doudna, J.A. (2020) CRISPR-CasPhi from huge phages is a hypercompact genome editor. *Science (New York, N.Y.)* **369**, 333-337.

Shen, Y., Ye, T., Li, Z., Kimutai, T.H., Song, H., Dong, X. and Wan, J. (2024) Exploiting viral vectors to deliver genome editing reagents in plants. *aBIOTECH* **5**, 247-261.

Sun, T., Liu, Q., Chen, X., Hu, F. and Wang, K. (2024a) Hi-TOM 2.0: an improved platform for high-throughput mutation detection. *Sci China Life Sci*.

Sun, Y., Hu, J., Hu, Z., Zhou, H., Gao, Y., Liu, Y., Ji, Y., Xu, G., Guo, Y., Zhang, Y., Tian, Y., Liu, X., Zhou, S., Liu, Y., Li, T., Li, C. and Wan, J. (2024b) Engineered and split an efficient hypercompact CRISPR-CasΦ genome editor in plants. *Plant Communications*.

Tang, N. and Ji, Q. (2024) Miniature CRISPR-Cas12 Systems: Mechanisms, Engineering, and Genome Editing Applications. *ACS Chemical Biology* **19**, 1399-1408.

Tang, X., Ren, Q., Yang, L., Bao, Y., Zhong, Z., He, Y., Liu, S., Qi, C., Liu, B., Wang, Y., Sretenovic, S., Zhang, Y., Zheng, X., Zhang, T., Qi, Y. and Zhang, Y. (2019) Single transcript unit CRISPR 2.0 systems for robust Cas9 and Cas12a mediated plant genome editing. *Plant biotechnology journal* **17**, 1431-1445.

Tang, X., Zheng, X., Qi, Y., Zhang, D., Cheng, Y., Tang, A., Voytas, D.F. and Zhang, Y. (2016) A Single Transcript CRISPR-Cas9 System for Efficient Genome Editing in Plants. *Mol Plant* **9**, 1088-1091.

Tuncel, A., Pan, C., Clem, J.S., Liu, D. and Qi, Y. (2025) CRISPR–Cas applications in agriculture and plant research. *Nature Reviews Molecular Cell Biology* **26**, 419-441.

Uranga, M., Aragones, V., Selma, S., Vazquez-Vilar, M., Orzaez, D. and Daros, J.A. (2021) Efficient Cas9 multiplex editing using unspaced sgRNA arrays engineering in a Potato virus X vector. *Plant J* **106**, 555-565.

Wu, J., Zhang, Y., Li, F., Zhang, X., Ye, J., Wei, T., Li, Z., Tao, X., Cui, F., Wang, X., Zhang, L., Yan, F., Li, S., Liu, Y., Li, D., Zhou, X. and Li, Y. (2024) Plant virology in the 21st century in China: Recent advances and future directions. *Journal of Integrative Plant Biology* **66**, 579-622.

Wu, L., Yang, J., Gu, Y., Wang, Q., Zhang, Z., Guo, H., Zhao, L., Zhang, H. and Gu, L. (2025) Bamboo mosaic virus‐mediated transgene‐free genome editing in bamboo. *New Phytologist* **245**, 1810-1816.

Wu, Z., Zhang, Y., Yu, H., Pan, D., Wang, Y., Wang, Y., Li, F., Liu, C., Nan, H., Chen, W. and Ji, Q. (2021) Programmed genome editing by a miniature CRISPR-Cas12f nuclease. *Nat Chem Biol* **17**, 1132-1138.

Yang, C.D., Liao, J.T., Lai, C.Y., Jong, M.H., Liang, C.M., Lin, Y.L., Lin, N.S., Hsu, Y.H. and Liang, S.M. (2007) Induction of protective immunity in swine by recombinant bamboo mosaic virus expressing foot-and-mouth disease virus epitopes. *BMC Biotechnol* **7**, 62.

Ye, Z., Zhang, Y., He, S., Li, S., Luo, L., Zhou, Y., Tan, J. and Wan, J. (2024) Efficient genome editing in rice with miniature Cas12f variants. *aBIOTECH* **5**, 184-188.

Zhan, X., Lu, Y., Zhu, J.K. and Botella, J.R. (2021) Genome editing for plant research and crop improvement. *J Integr Plant Biol* **63**, 3-33.
