# Supplementary material for: BaMV‐Vectored Compact AsCas12f1‐HKRA Enables Transgene‐Free Genome Editing in Moso Bamboo ( Phyllostachys edulis )
Source: Plant Biotechnol J. 2025 Dec 2;24(4):2220–2. doi: 10.1111/pbi.70474 (PMC13140604; doi:10.1111/pbi.70474)
Supplement: Supplementary file 2 — Appendix S2: pbi70474‐sup‐0002‐AppendixS2.docx. Supplemental Tables 1‐3. [file PBI-24-2220-s002.docx]

**Supplemental Table 1.** Target sites used in this study.

| **Target site** | **Nuclease** | **Targeted sequence (5'→3')** | **Target plants** | **Targeted gene ID** |
| --- | --- | --- | --- | --- |
| NbPDS | SpCas9 | TTGGTAGTAGCGACTCCATGGGG | *N. benthamiana* | (NbPDS-A) Niben101Scf01283Ctg022;  (NbPDS-B) Niben101Scf14708Ctg002 |
|  | AsCas12f1 | TTGGTAGTAGCGACTCCATGGGG |  |  |
|  | Cas12j | TTGGTAGTAGCGACTCCATGG |  |  |
| NbPSY | AsCas12f1 | TTGAGCTTGTTGAGTGAAGCATA | *N. benthamiana* | (NbPSY-A) Niben101Scf01959Ctg007;  (NbPSY-B) Niben101Scf04020Ctg004 |
| NbRDR6 | AsCas12f1 | TTGGCATTCTCAGCTAACCAGCT | 1. *benthamiana* | (NbRDR6-A) Niben101Scf12609Ctg016;  (NbRDR6-B) Niben101Scf03832Ctg041 |
| PhePDS-1 | AsCas12f1 | TTACGCCGTGGACTTGGTGCTCT | *P. edulis* | (PhePDS-A) PH02Gene05482;  (PhePDS-B) PH02Gene10404 |
| PhePDS-2 | AsCas12f1 | TTGGTATGAGACCGGGCTTCATA | *P. edulis* | (PhePDS-A) PH02Gene05482;  (PhePDS-B) PH02Gene10404 |
| PheRDR6 | SpCas9 | GGTGGCGGACAGGTTCTTGAGGG | *P. edulis* | (PheRDR6) PH02Gene35786 |
| PheRDR6 | AsCas12f1 | TTGAGGGTAACTTTTATGGATGA | *P. edulis* | (PheRDR6) PH02Gene35786 |
| Note: Conserved sgRNA loci target all copies of genes in host plants. The PAM sequences are highlighted in red. | | | | |

**Supplemental Table 2.** The sequences of the expression cassette for SpCas9, AsCas12f1, Cas12j, and crRNA.

| **Name** | **Sequence (5’ to 3’)** | **Note** |
| --- | --- | --- |
| BaMV-STU-Cas9-tRNA | ATGGAACGAGCTATACAAGGAAACGACGCTAGGGAACAAGCTAACAGTGAACGTTGGGATGGAGGATCAGGAGGTACCACTTCTCCCTTCAAACTTCCTGACGAAAGTCCGAGTTGGACTGAGTGGCGGCTACATAACGATGAGACGAATTCGAATCAAGATAATCCCCTTGGTTTCAAGGAAAGCTGGGGTTTCGGGAAAGTTGTATTTAAGAGATATCTCAGATACGACAGGACGGAAGCTTCACTGCACAGAGTCCTTGGATCTTGGACGGGAGATTCGGTTAACTATGCAGCATCTCGATTTTTCGGTTTCGACCAGATCGGATGTACCTATAGTATTCGGTTTCGAGGAGTTAGTATCACCGTTTCTGGAGGGTCGCGAACTCTTCAGCATCTCTGTGAGATGGCAATTCGGTCTAAGCAAGAACTGCTACAGCTTGCCCCAATCGAAGTGGAAAGTAATGTATCAAGAGGATGCCCTGAAGGTACTGAGACCTTCGAAAAAGAAAGCGAGggcagtggagagggcagaggaagtctgctaacatgcggtgacgtcgaggagaatcctggcccaATGGACTACAAAGACCATGACGGTGATTATAAAGATCATGACATCGACTACAAGGATGACGATGACAAGatggctcctaagaagaagcggaaggttggtattcacggggtgcctgcggctgacaagaagtactccatcggcctcgacatcggcaccaacagcgtcggctgggcggtgatcaccgacgagtacaaggtcccgtccaagaagttcaaggtcctgggcaacaccgaccgccactccatcaagaagaacctcatcggcgccctcctcttcgactccggcgagacggcggaggcgacccgcctcaagcgcaccgcccgccgccgctacacccgccgcaagaaccgcatctgctacctccaggagatcttctccaacgagatggcgaaggtcgacgactccttcttccaccgcctcgaggagtccttcctcgtggaggaggacaagaagcacgagcgccaccccatcttcggcaacatcgtcgacgaggtcgcctaccacgagaagtaccccactatctaccaccttcgtaagaagcttgttgactctactgataaggctgatcttcgtctcatctaccttgctctcgctcacatgatcaagttccgtggtcacttccttatcgagggtgaccttaaccctgataactccgacgtggacaagctcttcatccagctcgtccagacctacaaccagctcttcgaggagaaccctatcaacgcttccggtgtcgacgctaaggcgatcctttccgctaggctctccaagtccaggcgtctcgagaacctcatcgcccagctccctggtgagaagaagaacggtcttttcggtaacctcatcgctctctccctcggtctgacccctaacttcaagtccaacttcgacctcgctgaggacgctaagcttcagctctccaaggatacctacgacgatgatctcgacaacctcctcgctcagattggagatcagtacgctgatctcttccttgctgctaagaacctctccgatgctatcctcctttcggatatccttagggttaacactgagatcactaaggctcctctttctgcttccatgatcaagcgctacgacgagcaccaccaggacctcaccctcctcaaggctcttgttcgtcagcagctccccgagaagtacaaggagatcttcttcgaccagtccaagaacggctacgccggttacattgacggtggagctagccaggaggagttctacaagttcatcaagccaatccttgagaagatggatggtactgaggagcttctcgttaagcttaaccgtgaggacctccttaggaagcagaggactttcgataacggctctatccctcaccagatccaccttggtgagcttcacgccatccttcgtaggcaggaggacttctaccctttcctcaaggacaaccgtgagaagatcgagaagatccttactttccgtattccttactacgttggtcctcttgctcgtggtaactcccgtttcgcttggatgactaggaagtccgaggagactatcaccccttggaacttcgaggaggttgttgacaagggtgcttccgcccagtccttcatcgagcgcatgaccaacttcgacaagaacctccccaacgagaaggtcctccccaagcactccctcctctacgagtacttcacggtctacaacgagctcaccaaggtcaagtacgtcaccgagggtatgcgcaagcctgccttcctctccggcgagcagaagaaggctatcgttgacctcctcttcaagaccaaccgcaaggtcaccgtcaagcagctcaaggaggactacttcaagaagatcgagtgcttcgactccgtcgagatcagcggcgttgaggaccgtttcaacgcttctctcggtacctaccacgatctcctcaagatcatcaaggacaaggacttcctcgacaacgaggagaacgaggacatcctcgaggacatcgtcctcactcttactctcttcgaggatagggagatgatcgaggagaggctcaagacttacgctcatctcttcgatgacaaggttatgaagcagctcaagcgtcgccgttacaccggttggggtaggctctcccgcaagctcatcaacggtatcagggataagcagagcggcaagactatcctcgacttcctcaagtctgatggtttcgctaacaggaacttcatgcagctcatccacgatgactctcttaccttcaaggaggatattcagaaggctcaggtgtccggtcagggcgactctctccacgagcacattgctaaccttgctggttcccctgctatcaagaagggcatccttcagactgttaaggttgtcgatgagcttgtcaaggttatgggtcgtcacaagcctgagaacatcgtcatcgagatggctcgtgagaaccagactacccagaagggtcagaagaactcgagggagcgcatgaagaggattgaggagggtatcaaggagcttggttctcagatccttaaggagcaccctgtcgagaacacccagctccagaacgagaagctctacctctactacctccagaacggtagggatatgtacgttgaccaggagctcgacatcaacaggctttctgactacgacgtcgaccacattgttcctcagtctttccttaaggatgactccatcgacaacaaggtcctcacgaggtccgacaagaacaggggtaagtcggacaacgtcccttccgaggaggttgtcaagaagatgaagaactactggaggcagcttctcaacgctaagctcattacccagaggaagttcgacaacctcacgaaggctgagaggggtggcctttccgagcttgacaaggctggtttcatcaagaggcagcttgttgagacgaggcagattaccaagcacgttgctcagatcctcgattctaggatgaacaccaagtacgacgagaacgacaagctcatccgcgaggtcaaggtgatcaccctcaagtccaagctcgtctccgacttccgcaaggacttccagttctacaaggtccgcgagatcaacaactaccaccacgctcacgatgcttaccttaacgctgtcgttggtaccgctcttatcaagaagtaccctaagcttgagtccgagttcgtctacggtgactacaaggtctacgacgttcgtaagatgatcgccaagtccgagcaggagatcggcaaggccaccgccaagtacttcttctactccaacatcatgaacttcttcaagaccgagatcaccctcgccaacggcgagatccgcaagcgccctcttatcgagacgaacggtgagactggtgagatcgtttgggacaagggtcgcgacttcgctactgttcgcaaggtcctttctatgcctcaggttaacatcgtcaagaagaccgaggtccagaccggtggcttctccaaggagtctatccttccaaagagaaactcggacaagctcatcgctaggaagaaggattgggaccctaagaagtacggtggtttcgactcccctactgtcgcctactccgtcctcgtggtcgccaaggtggagaagggtaagtcgaagaagctcaagtccgtcaaggagctcctcggcatcaccatcatggagcgctcctccttcgagaagaacccgatcgacttcctcgaggccaagggctacaaggaggtcaagaaggacctcatcatcaagctccccaagtactctcttttcgagctcgagaacggtcgtaagaggatgctggcttccgctggtgagctccagaagggtaacgagcttgctcttccttccaagtacgtgaacttcctctacctcgcctcccactacgagaagctcaagggttcccctgaggataacgagcagaagcagctcttcgtggagcagcacaagcactacctcgacgagatcatcgagcagatctccgagttctccaagcgcgtcatcctcgctgacgctaacctcgacaaggtcctctccgcctacaacaagcaccgcgacaagcccatccgcgagcaggccgagaacatcatccacctcttcacgctcacgaacctcggcgcccctgctgctttcaagtacttcgacaccaccatcgacaggaagcgttacacgtccaccaaggaggttctcgacgctactctcatccaccagtccatcaccggtctttacgagactcgtatcgacctttcccagcttggtggtgataagcgtcctgctgccaccaaaaaggccggacaggctaagaaaaagaagtagAACAAAGCACCAGTGGTCTAGTGGTAGAATAGTACCCTGCCACGGTACAGACCCGGGTTCGATTCCCGGCTGGTGCANNNNNNNNNNNNNNNNNNNNGTTTTAGAGCTAGAAATAGCAAGTTAAAATAAGGCTAGTCCGTTATCAACTTGAAAAAGTGGCACCGAGTCGGTGCAACAAAGCACCAGTGGTCTAGTGGTAGAATAGTACCCTGCCACGGTACAGACCCGGGTTCGATTCCCGGCTGGTGCA | p19-T2A-3xFlag-SV40NLS-SpCas9-NLS-tRNA-sgRNA-tRNA |
| BaMV-STU-Cas9 | ATGGAACGAGCTATACAAGGAAACGACGCTAGGGAACAAGCTAACAGTGAACGTTGGGATGGAGGATCAGGAGGTACCACTTCTCCCTTCAAACTTCCTGACGAAAGTCCGAGTTGGACTGAGTGGCGGCTACATAACGATGAGACGAATTCGAATCAAGATAATCCCCTTGGTTTCAAGGAAAGCTGGGGTTTCGGGAAAGTTGTATTTAAGAGATATCTCAGATACGACAGGACGGAAGCTTCACTGCACAGAGTCCTTGGATCTTGGACGGGAGATTCGGTTAACTATGCAGCATCTCGATTTTTCGGTTTCGACCAGATCGGATGTACCTATAGTATTCGGTTTCGAGGAGTTAGTATCACCGTTTCTGGAGGGTCGCGAACTCTTCAGCATCTCTGTGAGATGGCAATTCGGTCTAAGCAAGAACTGCTACAGCTTGCCCCAATCGAAGTGGAAAGTAATGTATCAAGAGGATGCCCTGAAGGTACTGAGACCTTCGAAAAAGAAAGCGAGggcagtggagagggcagaggaagtctgctaacatgcggtgacgtcgaggagaatcctggcccaATGGACTACAAAGACCATGACGGTGATTATAAAGATCATGACATCGACTACAAGGATGACGATGACAAGatggctcctaagaagaagcggaaggttggtattcacggggtgcctgcggctgacaagaagtactccatcggcctcgacatcggcaccaacagcgtcggctgggcggtgatcaccgacgagtacaaggtcccgtccaagaagttcaaggtcctgggcaacaccgaccgccactccatcaagaagaacctcatcggcgccctcctcttcgactccggcgagacggcggaggcgacccgcctcaagcgcaccgcccgccgccgctacacccgccgcaagaaccgcatctgctacctccaggagatcttctccaacgagatggcgaaggtcgacgactccttcttccaccgcctcgaggagtccttcctcgtggaggaggacaagaagcacgagcgccaccccatcttcggcaacatcgtcgacgaggtcgcctaccacgagaagtaccccactatctaccaccttcgtaagaagcttgttgactctactgataaggctgatcttcgtctcatctaccttgctctcgctcacatgatcaagttccgtggtcacttccttatcgagggtgaccttaaccctgataactccgacgtggacaagctcttcatccagctcgtccagacctacaaccagctcttcgaggagaaccctatcaacgcttccggtgtcgacgctaaggcgatcctttccgctaggctctccaagtccaggcgtctcgagaacctcatcgcccagctccctggtgagaagaagaacggtcttttcggtaacctcatcgctctctccctcggtctgacccctaacttcaagtccaacttcgacctcgctgaggacgctaagcttcagctctccaaggatacctacgacgatgatctcgacaacctcctcgctcagattggagatcagtacgctgatctcttccttgctgctaagaacctctccgatgctatcctcctttcggatatccttagggttaacactgagatcactaaggctcctctttctgcttccatgatcaagcgctacgacgagcaccaccaggacctcaccctcctcaaggctcttgttcgtcagcagctccccgagaagtacaaggagatcttcttcgaccagtccaagaacggctacgccggttacattgacggtggagctagccaggaggagttctacaagttcatcaagccaatccttgagaagatggatggtactgaggagcttctcgttaagcttaaccgtgaggacctccttaggaagcagaggactttcgataacggctctatccctcaccagatccaccttggtgagcttcacgccatccttcgtaggcaggaggacttctaccctttcctcaaggacaaccgtgagaagatcgagaagatccttactttccgtattccttactacgttggtcctcttgctcgtggtaactcccgtttcgcttggatgactaggaagtccgaggagactatcaccccttggaacttcgaggaggttgttgacaagggtgcttccgcccagtccttcatcgagcgcatgaccaacttcgacaagaacctccccaacgagaaggtcctccccaagcactccctcctctacgagtacttcacggtctacaacgagctcaccaaggtcaagtacgtcaccgagggtatgcgcaagcctgccttcctctccggcgagcagaagaaggctatcgttgacctcctcttcaagaccaaccgcaaggtcaccgtcaagcagctcaaggaggactacttcaagaagatcgagtgcttcgactccgtcgagatcagcggcgttgaggaccgtttcaacgcttctctcggtacctaccacgatctcctcaagatcatcaaggacaaggacttcctcgacaacgaggagaacgaggacatcctcgaggacatcgtcctcactcttactctcttcgaggatagggagatgatcgaggagaggctcaagacttacgctcatctcttcgatgacaaggttatgaagcagctcaagcgtcgccgttacaccggttggggtaggctctcccgcaagctcatcaacggtatcagggataagcagagcggcaagactatcctcgacttcctcaagtctgatggtttcgctaacaggaacttcatgcagctcatccacgatgactctcttaccttcaaggaggatattcagaaggctcaggtgtccggtcagggcgactctctccacgagcacattgctaaccttgctggttcccctgctatcaagaagggcatccttcagactgttaaggttgtcgatgagcttgtcaaggttatgggtcgtcacaagcctgagaacatcgtcatcgagatggctcgtgagaaccagactacccagaagggtcagaagaactcgagggagcgcatgaagaggattgaggagggtatcaaggagcttggttctcagatccttaaggagcaccctgtcgagaacacccagctccagaacgagaagctctacctctactacctccagaacggtagggatatgtacgttgaccaggagctcgacatcaacaggctttctgactacgacgtcgaccacattgttcctcagtctttccttaaggatgactccatcgacaacaaggtcctcacgaggtccgacaagaacaggggtaagtcggacaacgtcccttccgaggaggttgtcaagaagatgaagaactactggaggcagcttctcaacgctaagctcattacccagaggaagttcgacaacctcacgaaggctgagaggggtggcctttccgagcttgacaaggctggtttcatcaagaggcagcttgttgagacgaggcagattaccaagcacgttgctcagatcctcgattctaggatgaacaccaagtacgacgagaacgacaagctcatccgcgaggtcaaggtgatcaccctcaagtccaagctcgtctccgacttccgcaaggacttccagttctacaaggtccgcgagatcaacaactaccaccacgctcacgatgcttaccttaacgctgtcgttggtaccgctcttatcaagaagtaccctaagcttgagtccgagttcgtctacggtgactacaaggtctacgacgttcgtaagatgatcgccaagtccgagcaggagatcggcaaggccaccgccaagtacttcttctactccaacatcatgaacttcttcaagaccgagatcaccctcgccaacggcgagatccgcaagcgccctcttatcgagacgaacggtgagactggtgagatcgtttgggacaagggtcgcgacttcgctactgttcgcaaggtcctttctatgcctcaggttaacatcgtcaagaagaccgaggtccagaccggtggcttctccaaggagtctatccttccaaagagaaactcggacaagctcatcgctaggaagaaggattgggaccctaagaagtacggtggtttcgactcccctactgtcgcctactccgtcctcgtggtcgccaaggtggagaagggtaagtcgaagaagctcaagtccgtcaaggagctcctcggcatcaccatcatggagcgctcctccttcgagaagaacccgatcgacttcctcgaggccaagggctacaaggaggtcaagaaggacctcatcatcaagctccccaagtactctcttttcgagctcgagaacggtcgtaagaggatgctggcttccgctggtgagctccagaagggtaacgagcttgctcttccttccaagtacgtgaacttcctctacctcgcctcccactacgagaagctcaagggttcccctgaggataacgagcagaagcagctcttcgtggagcagcacaagcactacctcgacgagatcatcgagcagatctccgagttctccaagcgcgtcatcctcgctgacgctaacctcgacaaggtcctctccgcctacaacaagcaccgcgacaagcccatccgcgagcaggccgagaacatcatccacctcttcacgctcacgaacctcggcgcccctgctgctttcaagtacttcgacaccaccatcgacaggaagcgttacacgtccaccaaggaggttctcgacgctactctcatccaccagtccatcaccggtctttacgagactcgtatcgacctttcccagcttggtggtgataagcgtcctgctgccaccaaaaaggccggacaggctaagaaaaagaagtagNNNNNNNNNNNNNNNNNNNNGTTTTAGAGCTAGAAATAGCAAGTTAAAATAAGGCTAGTCCGTTATCAACTTGAAAAAGTGGCACCGAGTCGGTGC | p19-T2A-3xFlag-SV40NLS-SpCas9-NLS-sgRNA |
| BaMV-STU-AsCas12f1_HKRA_-tRNA | ATGGAACGAGCTATACAAGGAAACGACGCTAGGGAACAAGCTAACAGTGAACGTTGGGATGGAGGATCAGGAGGTACCACTTCTCCCTTCAAACTTCCTGACGAAAGTCCGAGTTGGACTGAGTGGCGGCTACATAACGATGAGACGAATTCGAATCAAGATAATCCCCTTGGTTTCAAGGAAAGCTGGGGTTTCGGGAAAGTTGTATTTAAGAGATATCTCAGATACGACAGGACGGAAGCTTCACTGCACAGAGTCCTTGGATCTTGGACGGGAGATTCGGTTAACTATGCAGCATCTCGATTTTTCGGTTTCGACCAGATCGGATGTACCTATAGTATTCGGTTTCGAGGAGTTAGTATCACCGTTTCTGGAGGGTCGCGAACTCTTCAGCATCTCTGTGAGATGGCAATTCGGTCTAAGCAAGAACTGCTACAGCTTGCCCCAATCGAAGTGGAAAGTAATGTATCAAGAGGATGCCCTGAAGGTACTGAGACCTTCGAAAAAGAAAGCGAGggcagtggagagggcagaggaagtctgctaacatgcggtgacgtcgaggagaatcctggcccaatggactataaggaccacgacggagactacaaggatcatgatattgattacaaagacgatgacgataagatggctcctaagaagaagcggaaggttggtattcacggggtgcctgcggctATGATCAAGGTGTACAGGTACGAGATCGTGAAGCCGCTCGACCTCGACTGGAAGGAGTTCGGCACCATCCTCAGGCAGCTCCAGCAGGAGACAAGGTTCGCCCTCAACAAGGCCACCCAGCTCGCCTGGGAGTGGATGGGCTTCTCCTCCGACTACAAGGACAACCACGGCGAGTACCCGAAGTCCAAGGACATCCTCGGCTACACCAACGTGCACGGCTACGCCTACCACACCATCAAGACCAAGGCCTACAGGCTCAACTCCGGCAACCTCTCCCAGACCATCAAGAGGGCCACCGACAGGTTCAAGGCCTACCAGAAGGAGATCCTCAGGGGCGACATGTCCATCCCGTCCTACAAGAGGGACCACCCGCTCGACCTCATCAAGGAGAACATCTCCGTGAACAGGATGAACCACGGCGACTACATCGCCTCCCTCTCCCTCCTCTCCAACCCGGCCAAGCAGGAGATGAACGTGAAGAGGAAGATCTCCGTGATCATCATCGTGAGGGGCGCCGGCAAGACCATCATGGACAGGATCCTCTCCGGCGAGTACCAGGTGTCCGCCTCCCAGATCATCCACAAGGACAGGAAGAACAAGTGGTACCTCAACATCTCCTACAGGTTCGAGCCGCAGACCAGGGTGCTCGACCTCAACAAGATCATGGGCATCGACCTCGGCGTGGCCGTGGCCGCGTACATGGCCTTCCAGCACACCCCGGCCAGGTACAAGCTCGAGGGCGGCGAGATCGAGAACTTCAGGAGGCAGGTGGAGTCCAGGAGGATCTCCATGCTCAGGCAGGGCAAGTACGCCGGCGGCGCCAGGGGCGGCCACGGCAGGGACAAGAGGATCAAGCCGATCGAGCAGCTCAGGGACAAGATCGCCAACTTCAGGGACACCACCAACCACAGGTACTCCAGGTACATCGTGGACATGGCCATCAAGGAGGGCTGCGGCACCATCCAGATGGAGGACCTCACCAACATCAGGGACATCGGCTCCAGGTTCCTCCAGAACTGGACCTACTACGACCTCCAGCAGAAGATCATCTACAAGGCCGAGGAGGCCGGCATCAAGGTGATCAAGATCGACCCGCAGTACACCTCCCAGAGGTGCTCCGAGTGCGGCAACATCGACTCCGGCAACAGGATCGGCCAGGCCATCTTCAAGTGCAGGGCCTGCGGCTACGAGGCCAACGCCGACTACAACGCCGCCAGGAACATCGCCATCCCGAACATCGACAAGATCATCGCCGAGTCCATCAAGaagcgtcctgctgccaccaaaaaggccggacaggctaagaaaaagaagtagAACAAAGCACCAGTGGTCTAGTGGTAGAATAGTACCCTGCCACGGTACAGACCCGGGTTCGATTCCCGGCTGGTGCATCGTCGGTTCAGCGACGATAAGCCGAGAAGTGCCAATAAAACTGTTAAGTGGTTTGGTAACGCTCGGTAAGGTCCGAAAGGAGAACCACTGAACNNNNNNNNNNNNNNNNNNNNAACAAAGCACCAGTGGTCTAGTGGTAGAATAGTACCCTGCCACGGTACAGACCCGGGTTCGATTCCCGGCTGGTGCA | p19-T2A-3xFlag-SV40NLS-AsCas12f1-HKRA-NLS-tRNA-sgRNA-tRNA |
| BaMV-STU-AsCas12f1_YHAM_-tRNA | ATGGAACGAGCTATACAAGGAAACGACGCTAGGGAACAAGCTAACAGTGAACGTTGGGATGGAGGATCAGGAGGTACCACTTCTCCCTTCAAACTTCCTGACGAAAGTCCGAGTTGGACTGAGTGGCGGCTACATAACGATGAGACGAATTCGAATCAAGATAATCCCCTTGGTTTCAAGGAAAGCTGGGGTTTCGGGAAAGTTGTATTTAAGAGATATCTCAGATACGACAGGACGGAAGCTTCACTGCACAGAGTCCTTGGATCTTGGACGGGAGATTCGGTTAACTATGCAGCATCTCGATTTTTCGGTTTCGACCAGATCGGATGTACCTATAGTATTCGGTTTCGAGGAGTTAGTATCACCGTTTCTGGAGGGTCGCGAACTCTTCAGCATCTCTGTGAGATGGCAATTCGGTCTAAGCAAGAACTGCTACAGCTTGCCCCAATCGAAGTGGAAAGTAATGTATCAAGAGGATGCCCTGAAGGTACTGAGACCTTCGAAAAAGAAAGCGAGggcagtggagagggcagaggaagtctgctaacatgcggtgacgtcgaggagaatcctggcccaatggactataaggaccacgacggagactacaaggatcatgatattgattacaaagacgatgacgataagatggctcctaagaagaagcggaaggttggtattcacggggtgcctgcggctATGATCAAGGTGTACAGGTACGAGATCGTGAAGCCGCTCGACCTCGACTGGAAGGAGTTCGGCACCATCCTCAGGCAGCTCCAGCAGGAGACAAGGTTCGCCCTCAACAAGGCCACCCAGCTCGCCTGGGAGTGGATGGGCTACTCCTCCGACTACAAGGACAACCACGGCGAGTACCCGAAGTCCAAGGACATCCTCGGCTACACCAACGTGCACGGCTACGCCTACCACACCATCAAGACCAAGGCCTACAGGCTCAACTCCGGCAACCTCTCCCAGACCATCAAGAGGGCCACCGACAGGTTCAAGGCCTACCAGAAGGAGATCCTCAGGGGCGACATGTCCATCCCGTCCTACAAGAGGGACATCCCGCTCGACCTCATCAAGGAGAACATCTCCGTGAACAGGATGAACCACGGCGACTACATCGCCTCCCTCTCCCTCCTCTCCAACCCGGCCAAGCAGGAGATGAACGTGAAGAGGAAGATCTCCGTGATCATCATCGTGAGGGGCGCCGGCAAGACCATCATGGACAGGATCCTCTCCGGCGAGTACCAGGTGCACGCCTCCCAGATCATCCACGACGACAGGAAGAACAAGTGGTACCTCAACATCTCCTACGACTTCGAGCCGCAGACCAGGGTGCTCGACCTCAACAAGATCATGGGCATCGACCTCGGCGTGGCCGTGGCCGCGTACATGGCCTTCCAGCACACCCCGGCCAGGTACAAGCTCGAGGGCGGCGAGATCGAGAACTTCAGGAGGCAGGTGGAGTCCAGGAGGATCTCCATGCTCAGGCAGGGCAAGTACGCCGGCGGCGCCAGGGGCGGCCACGGCAGGGACAAGAGGATCAAGCCGATCGAGCAGCTCAGGGACAAGATCGCCAACTTCAGGGACACCACCAACCACAGGTACTCCAGGTACATCGTGGACATGGCCATCAAGATGGGCTGCGGCACCATCCAGATGGAGGACCTCACCAACATCAGGGACATCGGCTCCAGGTTCCTCCAGAACTGGACCTACTACGACCTCCAGCAGAAGATCATCTACAAGGCCGAGGAGGCCGGCATCAAGGTGATCAAGATCGACCCGCAGTACACCTCCCAGAGGTGCTCCGAGTGCGGCAACATCGACTCCGGCAACAGGATCGGCCAGGCCATCTTCAAGTGCAGGGCCTGCGGCTACGAGGCCAACGCCGACTACAACGCCGCCAGGAACATCGCCATCCCGAACATCGACAAGATCATCGCCGAGTCCATCAAGaagcgtcctgctgccaccaaaaaggccggacaggctaagaaaaagaagtagAACAAAGCACCAGTGGTCTAGTGGTAGAATAGTACCCTGCCACGGTACAGACCCGGGTTCGATTCCCGGCTGGTGCATCGTCGGTTCAGCGACGATAAGCCGAGAAGTGCCAATAAAACTGTTAAGTGGTTTGGTAACGCTCGGTAAGGTCCGAAAGGAGAACCACTGAACNNNNNNNNNNNNNNNNNNNNAACAAAGCACCAGTGGTCTAGTGGTAGAATAGTACCCTGCCACGGTACAGACCCGGGTTCGATTCCCGGCTGGTGCA | p19-T2A-3xFlag-SV40NLS-AsCas12f1-YHAM-NLS-tRNA-sgRNA-tRNA |
| BaMV-STU-nCas12j2-tRNA | ATGGAACGAGCTATACAAGGAAACGACGCTAGGGAACAAGCTAACAGTGAACGTTGGGATGGAGGATCAGGAGGTACCACTTCTCCCTTCAAACTTCCTGACGAAAGTCCGAGTTGGACTGAGTGGCGGCTACATAACGATGAGACGAATTCGAATCAAGATAATCCCCTTGGTTTCAAGGAAAGCTGGGGTTTCGGGAAAGTTGTATTTAAGAGATATCTCAGATACGACAGGACGGAAGCTTCACTGCACAGAGTCCTTGGATCTTGGACGGGAGATTCGGTTAACTATGCAGCATCTCGATTTTTCGGTTTCGACCAGATCGGATGTACCTATAGTATTCGGTTTCGAGGAGTTAGTATCACCGTTTCTGGAGGGTCGCGAACTCTTCAGCATCTCTGTGAGATGGCAATTCGGTCTAAGCAAGAACTGCTACAGCTTGCCCCAATCGAAGTGGAAAGTAATGTATCAAGAGGATGCCCTGAAGGTACTGAGACCTTCGAAAAAGAAAGCGAGggcagtggagagggcagaggaagtctgctaacatgcggtgacgtcgaggagaatcctggcccaatggactataaggaccacgacggagactacaaggatcatgatattgattacaaagacgatgacgataagatggctcctaagaagaagcggaaggttggtattcacggggtgcctgcggctATGCCAAAGCCAGCCGTCGAGTCAGAGTTTTCAAAGGTTCTCAAGAAGCATTTTCCTGGAGAAAGGTTCCGGTCGAGCTACATGAAGAGAGGAGGAAAGATTCTAGCTGCTCAAGGTGAAGAAGCGGTGGTAGCTTATTTGCAAGGCAAGTCGGAAGAAGAGCCACCAAATTTCCAGCCGCCGGCCAAATGCCACGTCGTCACAAAGTCACGTGATTTTGCAGAGTGGCCAATCATGAAAGCTAGTGAAGCAATCCAGAGATATATTTATGCATTGTCGACAACTGAGAGGGCAGCGTGCAAGCCAGGAAAATCCTCGGAGAGCCATGCTGCGTGGTTTGCTGCTACTGGAGTAAGTAATCATGGCTACTCACATGTGCAAGGGCTGAACCTTATCTTCGACCACACCCTTGGTAGATACGACGGGGTGCTCAAAAAGGTGCAGTTGAGAAATGAAAAGGCGCGCGCCAGGCTGGCCGCCATCAACGCCGCCAGAGCCGCCGCGGGTCTACCTGAGATCAAGGCCGAAGAGGAAGAAGTCGCGACAAATGAGACTGGCCACCTCCTGCAGCCTCCTGGCATCAATCCATCATTCTATGTGTACCAGACAATTTCTCCGCAGGCGTACCGCCCCCGCGACGAGATTGTTCTTCCGCCCGAGTATGCCGGCTATGTGCGTGATCCTAATGCACCAATACCCCTTGGGGTTGTCAGGAACCGTTGCGACATTCAGAAAGGCTGCCCTGGTTATATCCCTGAATGGCAGAGGGAGGCAGGGACGGCCATCTCTCCAAAGACCGGCAAGGCAGTGACCGTCCCCGGCCTCTCCCCGAAAAAGAACAAGAGGATGAGGAGATACTGGAGGAGCGAGAAGGAAAAAGCGCAGGACGCCCTACTCGTCACCGTGCGGATCGGTACAGACTGGGTAGTCATCGACGTCCGCGGCCTGTTAAGGAATGCCAGGTGGCGGACTATTGCTCCAAAAGATATATCTCTCAATGCTCTCCTTGACCTCTTCACCGGAGATCCGGTGATTGATGTTCGCCGCAACATAGTGACGTTCACATACACCTTGGATGCTTGTGGCACCTACGCCAGGAAGTGGACACTCAAAGGCAAGCAGACCAAGGCGACGCTCGATAAGCTCACCGCAACTCAAACTGTGGCGCTGGTTGCAATTGACCTTGGGCAAACCAACCCCATATCAGCTGGAATTTCCCGTGTTACACAAGAAAATGGTGCGCTTCAATGTGAACCTCTGGATAGGTTTACTCTCCCTGATGATTTGTTAAAGGATATCTCTGCGTACCGGATTGCCTGGGATCGAAACGAAGAGGAACTTCGCGCTCGATCCGTAGAAGCCCTGCCAGAGGCCCAGCAGGCGGAAGTGCGCGCGTTGGATGGTGTCTCCAAAGAGACTGCCCGGACCCAGCTCTGCGCCGACTTCGGGCTCGACCCCAAGCGCTTACCATGGGACAAGATGTCGTCCAACACCACTTTCATCAGTGAGGCATTGTTGTCCAATAGTGTTTCCCGCGATCAGGTGTTCTTCACTCCCGCGCCGAAGAAGGGAGCAAAAAAAAAAGCGCCAGTGGAGGTGATGAGAAAGGACCGCACATGGGCAAGAGCATACAAGCCCAGGCTGTCTGTTGAAGCTCAGAAGCTGAAGAATGAGGCGCTGTGGGCTTTGAAAAGAACGTCTCCGGAGTACCTCAAGCTATCAAGACGGAAAGAAGAGCTGTGCCGTCGCAGCATAAATTATGTAATCGAGAAGACGCGAAGAAGAACTCAATGCCAAATTGTTATACCAGTTATTGAAGATCTGAATGTCCGGTTTTTTCACGGCAGTGGGAAGCGCCTGCCTGGGTGGGACAACTTCTTTACCGCAAAGAAGGAGAACCGCTGGTTCATACAGGGCCTGCACAAGGCGTTCTCGGATTTACGTACGCATCGTAGCTTCTACGTGTTTGAGGTACGGCCGGAGCGCACAAGTATCACCTGCCCCAAATGTGGGCATTGTGAGGTTGGCAACCGGGATGGTGAGGCGTTCCAGTGCCTCAGTTGTGGTAAGACGTGCAACGCAGACCTTGATGTCGCCACCCACAACCTGACGCAAGTAGCTCTCACCGGCAAAACTATGCCCAAAAGGGAGGAGCCGAGGGACGCGCAAGGAACGGCGCCTGCTCGGAAGACAAAAAAGGCCAGCAAGTCAAAAGCTCCGCCGGCCGAGCGGGAGGACCAAACACCGGCGCAGGAGCCTTCTCAGACGTCGAGCGGATCAGGTAAGAGGCCTGCCGCAACAAAGAAGGCCGGTCAAGCCAAGAAGAAGAAGtagAACAAAGCACCAGTGGTCTAGTGGTAGAATAGTACCCTGCCACGGTACAGACCCGGGTTCGATTCCCGGCTGGTGCACAACGATTGCCCCTtACGAGGGGACNNNNNNNNNNNNNNNNNNAACAAAGCACCAGTGGTCTAGTGGTAGAATAGTACCCTGCCACGGTACAGACCCGGGTTCGATTCCCGGCTGGTGCA | p19-T2A-3xFlag-SV40NLS-nCas12j2-NLS-tRNA-sgRNA-tRNA |
| BaMV-STU-vCas12j2-tRNA | ATGGAACGAGCTATACAAGGAAACGACGCTAGGGAACAAGCTAACAGTGAACGTTGGGATGGAGGATCAGGAGGTACCACTTCTCCCTTCAAACTTCCTGACGAAAGTCCGAGTTGGACTGAGTGGCGGCTACATAACGATGAGACGAATTCGAATCAAGATAATCCCCTTGGTTTCAAGGAAAGCTGGGGTTTCGGGAAAGTTGTATTTAAGAGATATCTCAGATACGACAGGACGGAAGCTTCACTGCACAGAGTCCTTGGATCTTGGACGGGAGATTCGGTTAACTATGCAGCATCTCGATTTTTCGGTTTCGACCAGATCGGATGTACCTATAGTATTCGGTTTCGAGGAGTTAGTATCACCGTTTCTGGAGGGTCGCGAACTCTTCAGCATCTCTGTGAGATGGCAATTCGGTCTAAGCAAGAACTGCTACAGCTTGCCCCAATCGAAGTGGAAAGTAATGTATCAAGAGGATGCCCTGAAGGTACTGAGACCTTCGAAAAAGAAAGCGAGggcagtggagagggcagaggaagtctgctaacatgcggtgacgtcgaggagaatcctggcccaATGGATTACAAAGATCACGATGGTGACTACAAAGACCACGATATCGACTACAAGGACGACGATGACAAGATGGCGCCAAAGAAGAAGCGTAAGGTCATGCCAAAGCCTGCTGTTGAGTCTGAGTTCAGCAAGGTGCTGAAGAAGCACTTCCCTGGAGAGAGGTTCAGGTCCAGTTACATGAAGCGCGGAGGCAAGATACTTGCTGCTCAGGGTGAAGAGGCTGTTGTGGCGTATCTGCAGGGCAAGAGCGAGGAGGAGCCACCTAACTTCCAGCCGCCTGCCAAGTGCCACGTCGTGACCAAGTCCAGGGATTTCGCCGAGTGGCCAATCATGAAGGCCTCAGAGGCGATCCAGAGGTACATCTACGCTCTTTCTACCACCGAGCGCGCAGCGTGCAAACCGGGCAAGTCATCCGAGAGTCATGCGGCGTGGTTCGCTGCGACAGGCGTCTCGAATCACGGCTACTCACATGTTCAAGGCCTTAACCTCATCTTCGACCACACACTTGGCCGCTACGATGGCGTGCTCAAGAAGGTTCAGCTCCGCAACGAGAAGGCTGGCTCATCCGGCGAGGAGGAGGTGGCAACCAATGAGACGGGCCATCTGTTGCAGCCTCCGGGCATCAATCCGAGCTTCTACGTCTACCAGACAATCTCGCCTCAGGCCTACAGACCTAGAGACGAGATCGTGCTGCCACCAGAGTACGCGGGATACGTGAGAGATCCGAACGCGCCAATTCCATTGGGAGTGGTCCGCAACCGGTGCGATATCCAGAAGGGATGTCCAGGTTATATACCTGAGTGGCAAAGGGAGGCGGGCACCGCAATCTCTCCAAAGACCGGTAAGGCAGTGACAGTGCCTGGCCTGTCGCCTAAGAAGAACAAGCGCATGAGGCGGTACTGGAGAAGCGAGAAAGAGAAGGCTCAAGACGCTCTTCTTGTGACCGTGAGAATCGGCACAGATTGGGTCGTGATTGACGTGAGAGGCCTCTTGAGAAACGCGCGCTGGAGAACAATCGCTCCGAAGGACATCAGCTTGAACGCGTTGCTCGACCTCTTCACAGGTGATCCGGTGATCGACGTGCGGAGAAACATCGTCACGTTCACGTACACGTTGGATGCTTGCGGCACGTATGCGCGTAAGTGGACACTGAAGGGCAAGCAGACCAAGGCTACGCTGGACAAGCTGACTGCTACGCAGACTGTCGCCTTGGTCGCCATCGATCTCGGACAGACCAATCCGATCAGCGCGGGTATCTCCAGGGTCACGCAAGAGAACGGCGCTCTCCAGTGTGAGCCACTTGACCGCTTTACCTTGCCGGACGATCTGCTGAAAGACATCAGCGCCTACAGAATCGCGTGGGATCGCAACGAGGAGGAGCTCAGGGCCAGGTCAGTCGAGGCTCTGCCTGAAGCTCAGCAGGCGGAAGTCAGGGCCTTGGACGGTGTCTCGAAAGAGACCGCAAGAACACAACTGTGCGCTGACTTTGGCCTGGACCCAAAGAGGCTGCCTTGGGATAAGATGTCGTCCAACACCACCTTCATCAGCGAGGCACTGCTCTCCAACTCCGTGAGCAGGGATCAGGTCTTCTTCACGCCAGCACCAAAGAAGGGTGCGAAGAAGAAAGCGCCAGTCGAAGTCATGCGCAAAGACAGAACATGGGCCAGGGCGTACAAGCCGAGACTCTCTGTTGAGGCACAGAAGCTCAAGAACGAGGCCTTGTGGGCACTCAAGCGCACTTCACCGGAGTATCTGAAGCTCTCGAGGCGGAAAGAAGAGCTGTGTCGGAGAAGCATCAACTACGTCATCGAGAAGACCAGACGCAGAACTCAGTGCCAGATCGTGATCCCTGTGATCGAAGACCTCAACGTGAGGTTCTTCCACGGCAGCGGAAAGAGGCTTCCAGGCTGGGATAACTTCTTCACAGCCAAGAAAGAGAACAGGTGGTTCATCCAGGGCCTGCATAAGGCTTTCAGCGACCTCAGAACTCACAGGTCTTTCTACGTGTTCGAGGTGAGACCGGAGAGAACCTCAATCACCTGTCCGAAGTGCGGCCATTGTGAGGTGGGCAACAGGGACGGTGAGGCGTTTCAGTGCCTCAGCTGCGGCAAGACATGCAACGCAGACCTGGACGTTGCCACACATAACCTCACACAAGTTGCACTGACTGGCAAGACAATGCCTAAGAGGGAAGAACCGCGCGATGCGCAGGGAACAGCACCAGCCAGAAAGACTAAGAAGGCCAGCAAGAGCAAGGCACCTCCAGCTGAACGCGAAGACCAAACACCAGCTCAAGAGCCATCACAAACTAGCGGATCAGGTAAGAGGCCTGCCGCAACAAAGAAGGCCGGTCAAGCCAAGAAGAAGAAGtagAACAAAGCACCAGTGGTCTAGTGGTAGAATAGTACCCTGCCACGGTACAGACCCGGGTTCGATTCCCGGCTGGTGCACAACGATTGCCCCTtACGAGGGGACNNNNNNNNNNNNNNNNNNAACAAAGCACCAGTGGTCTAGTGGTAGAATAGTACCCTGCCACGGTACAGACCCGGGTTCGATTCCCGGCTGGTGCA | p19-T2A-3xFlag-SV40NLS-vCas12j2-NLS-tRNA-sgRNA-tRNA |
| BaMV-STU-enCas12j8-tRNA | ATGGAACGAGCTATACAAGGAAACGACGCTAGGGAACAAGCTAACAGTGAACGTTGGGATGGAGGATCAGGAGGTACCACTTCTCCCTTCAAACTTCCTGACGAAAGTCCGAGTTGGACTGAGTGGCGGCTACATAACGATGAGACGAATTCGAATCAAGATAATCCCCTTGGTTTCAAGGAAAGCTGGGGTTTCGGGAAAGTTGTATTTAAGAGATATCTCAGATACGACAGGACGGAAGCTTCACTGCACAGAGTCCTTGGATCTTGGACGGGAGATTCGGTTAACTATGCAGCATCTCGATTTTTCGGTTTCGACCAGATCGGATGTACCTATAGTATTCGGTTTCGAGGAGTTAGTATCACCGTTTCTGGAGGGTCGCGAACTCTTCAGCATCTCTGTGAGATGGCAATTCGGTCTAAGCAAGAACTGCTACAGCTTGCCCCAATCGAAGTGGAAAGTAATGTATCAAGAGGATGCCCTGAAGGTACTGAGACCTTCGAAAAAGAAAGCGAGggcagtggagagggcagaggaagtctgctaacatgcggtgacgtcgaggagaatcctggcccaatggactataaggaccacgacggagactacaaggatcatgatattgattacaaagacgatgacgataagATGGCCCCAAAGAAGAAGCGCAAGGTCATCAAGCCAACCGTCTCCCAGTTCCTGACACCTGGCTTTAAGCTGATCAGAAACCACAGCAGGACAGCCGGCCTGAAGCTGAAGAATGAGGGAGAAGAAGCTTGTAAAAAATTCGTGCGGGAGAACGAGATCCCTAAAGACGAGTGCCCCAACTTCCAGGGCGGCCCTGCCATCGCTAACATCATCGCCAAGAGCAGAGAATTTACCGAGTGGGAGATCTACCAGTCTAGCCTGGCTATTCAGGAGGTGATCTTCACCCTGCCTAAAGATAAGCTGCCTGAACCTATCCTGAAGGAAGAATGGAGAGCCCAATGGCTGTCTGAGCACGGCCTGGACACCGTTCCTTACAAGGAGGCCcagGGACTCAACCTGATCATCAAGAACGCCGTGAATACCTACAAGGGCGTGCAGGTGAAGGTGGACAACAAGAACAAGAACAACCTGGCCAAGATCAATAGAAAGAATGAGATCGCCAAGCTGAACGGAGAGCAGGAGATCAGCTTCGAGGAAATCAAGGCCTTTGACGACAAGGGATACCTGCTGCAGAAGCCCAGCCCTAACAAGTCCATCTATTGCTACCAAcagGTGTCCCCAAAGCCCTTCATCACCAGCAAATACCACAACGTGAACCTGCCAGAGGAGTACATCGGCTACTACAGAAAGTCTAATGAACCCATCGTGTCTCCCTACCAGTTCGATAGACTGCGGATCCCCATCGGCGAGCCTGGATATGTGCCTAAGTGGCAGTACACCTTCCTGTCTAAGAAGGAAAACAAACGGAGAAAGCTTTCTAAAAGGATCAAGAATGTAAGCCCCATCCTGGGCATCATTTGTATCAAAAAGGACTGGTGCGTGTTCGACATGCGGGGACTGCTGAGAACCAACCATTGGAAGAAGTACCACAAGCCTACAGATAGCATCAATGATCTGTTCGACTATTTTACAGGCGACCCCGTGATCGACACAAAGGCCAATGTGGTCAGATTCCGGTACAAGATGGAAAATGGCATCGTGAATTACAAACCCGTGCGCGAAAAAAAGGGCAAAGAGCTGCTGGAAAACATTTGCGATCAAAACGGTAGCTGCAAGCTGGCTACAGTCGATGTGGGCCAGAACAACCCCGTGGCCATCGGCCTCTTCGAGCTGAAGAAGGTGAACGGCGAGCTGACCAAGACACTGATCAGCAGACACCCAACCCCTATCGACTTCTGCAACAAGATCACCGCCTACCGGGAAAGATACGACAAGCTGGAAAGCTCTATTAAGCTGGACGCCATCAAGCAGCTGACCAGCGAGCAGAAAATCGAGGTGGACAACTACAACAACAACTTCACCCCTCAGAACACCAAGCAGATCGTGTGCAGCAAGCTGAACATCAACCCCAACGACCTGCCTTGGGACAAGATGATCAGCGGCACACACTTCATCTCTGAAAAGGCACAGGTGAGCAACAAGTCTGAGATCTACTTCACCTCCACCGACAAGGGCAAGACAAAGGACGTGATGAAGAGCGACTACAAGTGGTTTCAAGACTACAAGCCTAAGCTGTCTAAGGAAGTGCGAGACGCCCTGTCAGATATCGAGTGGCGGCTGAGAAGAGAGAGCCTGGAATTCAACAAACTGAGCAAAAGCAGAGAGCAGGACGCCAGACAGCTGGCCAACTGGATCAGCAGCATGTGCGATGTGATCGGCATCGAGAACCTGGTGAAAAAGAACAACTTCTTCGGCGGCAGCGGCAAACGGGAACCTGGCTGGGATAATTTCTACAAGCCTAAGAAGGAAAACCGGTGGTGGATCAACGCCATCCACAAGGCTCTGACTGAACTGTCCCAGAACAAGGGCAAGAGAGTGATCTTACTGCCTGCTATGCGGACCAGCATCACCTGTCCTAAGTGTAAATACTGTGATAGCAAGAACAGAAACGGCGAGAAGTTCAACTGCCTGAAGTGCGGCATCGAGCTGAACGCCGACATTGATGTTGCTACAGAGAATCTGGCCACCGTGGCCATTACCGCGCAGTCCATGCCTAAGCCCACCTGCGAGAGAAGCGGCGATGCCAAGAAACCGGTCCGCGCCAGAAAGGCCAAAGCCCCTGAGTTTCACGACAAGCTCGCTCCTAGCTACACAGTCGTGCTGAGAGAGGCCGTGGGATCAGGTAAGAGGCCTGCCGCAACAAAGAAGGCCGGTCAAGCCAAGAAGAAGAAGtgaAACAAAGCACCAGTGGTCTAGTGGTAGAATAGTACCCTGCCACGGTACAGACCCGGGTTCGATTCCCGGCTGGTGCACTTTCAAGACTAATAGATTGCTCCTTACGAGGAGACNNNNNNNNNNNNNNNNNNAACAAAGCACCAGTGGTCTAGTGGTAGAATAGTACCCTGCCACGGTACAGACCCGGGTTCGATTCCCGGCTGGTGCA | p19-T2A-3xFlag-SV40NLS-enCas12j8-NLS-tRNA-sgRNA-tRNA |
| BaMV-STU-AsCas12f1_HKRA_-tRNA-CP1 | ATGTCCGGAACTGGTACAGGTACAGGGCGGGGGACGGGAACGGGGACTGGTGGCGCTGGAGGCACAGGCGGCACAGGCGGTGGAGGAGTTGGCAGAACCCAACAGGCTGCAAGCCAACCCTGGGAGGCGAAGTTCACTAAAGACGACCTGGCCGCAATTGAACCAAAACCCGCCTCAGCAAACGTGCCAAACACGAAGCAGTGGACTAACATCCAAGCCGGGCTTGTCAAAGCCGGGGCCACGGATGCAAACTTCATGAAAGTGCTGCTTGGCCTCAGCCTAGAAGCTTTCGACAGGGGTTCATCAGAAGCCACCACATGGGACGGCACCACTGAGGGCGTGGAACACCGTGCAGCGGCGAACGCGATTAAGGAAGCAAACTGCCCAATACACAAGGTTACCTATTACCTGGCCAAACCGACGTTCGCCATCAGGCAGTCCAAAAACCTTCCCCCAGCCAACTACGCAAAGAAAAATGTGCCGTCACAATACAAATGGTGCGCGTTCGACGCGTTTGACGGATTATACGACCCGACCTGCTTAGCCTCAGAGCTGCCCTATGACGCCCCCTCTGAAATAGACCGGATGGCATATGCCACTTTCAAAACCATACAGATCAAGACCGCTAATGATCAAAAGGGGTTCAACCTCAACTACAACCCCAACGTCACTCAAGCACGGCTCCCAAACACACCTCTGCCCGCCCTCCCTGAACCAGCATCAGACggcagtggagagggcagaggaagtctgctaacatgcggtgacgtcgaggagaatcctggcccaATGGAACGAGCTATACAAGGAAACGACGCTAGGGAACAAGCTAACAGTGAACGTTGGGATGGAGGATCAGGAGGTACCACTTCTCCCTTCAAACTTCCTGACGAAAGTCCGAGTTGGACTGAGTGGCGGCTACATAACGATGAGACGAATTCGAATCAAGATAATCCCCTTGGTTTCAAGGAAAGCTGGGGTTTCGGGAAAGTTGTATTTAAGAGATATCTCAGATACGACAGGACGGAAGCTTCACTGCACAGAGTCCTTGGATCTTGGACGGGAGATTCGGTTAACTATGCAGCATCTCGATTTTTCGGTTTCGACCAGATCGGATGTACCTATAGTATTCGGTTTCGAGGAGTTAGTATCACCGTTTCTGGAGGGTCGCGAACTCTTCAGCATCTCTGTGAGATGGCAATTCGGTCTAAGCAAGAACTGCTACAGCTTGCCCCAATCGAAGTGGAAAGTAATGTATCAAGAGGATGCCCTGAAGGTACTGAGACCTTCGAAAAAGAAAGCGAGggcagtggagagggcagaggaagtctgctaacatgcggtgacgtcgaggagaatcctggcccaatggactataaggaccacgacggagactacaaggatcatgatattgattacaaagacgatgacgataagatggctcctaagaagaagcggaaggttggtattcacggggtgcctgcggctATGATCAAGGTGTACAGGTACGAGATCGTGAAGCCGCTCGACCTCGACTGGAAGGAGTTCGGCACCATCCTCAGGCAGCTCCAGCAGGAGACAAGGTTCGCCCTCAACAAGGCCACCCAGCTCGCCTGGGAGTGGATGGGCTTCTCCTCCGACTACAAGGACAACCACGGCGAGTACCCGAAGTCCAAGGACATCCTCGGCTACACCAACGTGCACGGCTACGCCTACCACACCATCAAGACCAAGGCCTACAGGCTCAACTCCGGCAACCTCTCCCAGACCATCAAGAGGGCCACCGACAGGTTCAAGGCCTACCAGAAGGAGATCCTCAGGGGCGACATGTCCATCCCGTCCTACAAGAGGGACCACCCGCTCGACCTCATCAAGGAGAACATCTCCGTGAACAGGATGAACCACGGCGACTACATCGCCTCCCTCTCCCTCCTCTCCAACCCGGCCAAGCAGGAGATGAACGTGAAGAGGAAGATCTCCGTGATCATCATCGTGAGGGGCGCCGGCAAGACCATCATGGACAGGATCCTCTCCGGCGAGTACCAGGTGTCCGCCTCCCAGATCATCCACAAGGACAGGAAGAACAAGTGGTACCTCAACATCTCCTACAGGTTCGAGCCGCAGACCAGGGTGCTCGACCTCAACAAGATCATGGGCATCGACCTCGGCGTGGCCGTGGCCGCGTACATGGCCTTCCAGCACACCCCGGCCAGGTACAAGCTCGAGGGCGGCGAGATCGAGAACTTCAGGAGGCAGGTGGAGTCCAGGAGGATCTCCATGCTCAGGCAGGGCAAGTACGCCGGCGGCGCCAGGGGCGGCCACGGCAGGGACAAGAGGATCAAGCCGATCGAGCAGCTCAGGGACAAGATCGCCAACTTCAGGGACACCACCAACCACAGGTACTCCAGGTACATCGTGGACATGGCCATCAAGGAGGGCTGCGGCACCATCCAGATGGAGGACCTCACCAACATCAGGGACATCGGCTCCAGGTTCCTCCAGAACTGGACCTACTACGACCTCCAGCAGAAGATCATCTACAAGGCCGAGGAGGCCGGCATCAAGGTGATCAAGATCGACCCGCAGTACACCTCCCAGAGGTGCTCCGAGTGCGGCAACATCGACTCCGGCAACAGGATCGGCCAGGCCATCTTCAAGTGCAGGGCCTGCGGCTACGAGGCCAACGCCGACTACAACGCCGCCAGGAACATCGCCATCCCGAACATCGACAAGATCATCGCCGAGTCCATCAAGaagcgtcctgctgccaccaaaaaggccggacaggctaagaaaaagaagtagAACAAAGCACCAGTGGTCTAGTGGTAGAATAGTACCCTGCCACGGTACAGACCCGGGTTCGATTCCCGGCTGGTGCATCGTCGGTTCAGCGACGATAAGCCGAGAAGTGCCAATAAAACTGTTAAGTGGTTTGGTAACGCTCGGTAAGGTCCGAAAGGAGAACCACTGAACNNNNNNNNNNNNNNNNNNNNAACAAAGCACCAGTGGTCTAGTGGTAGAATAGTACCCTGCCACGGTACAGACCCGGGTTCGATTCCCGGCTGGTGCAACGTTGCATGATCGTAAAACATGCCCGGCTTACCGTGAGCCGCCTTTGAAAGAAAGGTTTACACGGATCCTGTTGTGTCACGCACGTACCTAACTTGTGTCAGCAGAATAAAGACCTTTTGGTTTCTACAGTTTTTTCCA | CP-T2A-p19-T2A-3xFlag-SV40NLS-AsCas12f1-HKRA-NLS-tRNA-sgRNA-tRNA-3’UTR |
| BaMV-STU-AsCas12f1_HKRA_-tRNA-CP2 | ATGTCCGGAACTGGTACAGGTACAGGGCGGGGGACGGGAACGGGGACTGGTGGCGCTGGAGGCACAGGCGGCACAGGCGGTGGAGGAGTTGGCAGAACCCAACAGGCTGCAAGCCAACCCTGGGAGGCGAAGTTCACTAAAGACGACCTGGCCGCAATTGAACCAAAACCCGCCTCAGCAAACGTGCCAAACACGAAGCAGTGGACTAACATCCAAGCCGGGCTTGTCAAAGCCGGGGCCACGGATGCAAACTTCATGAAAGTGCTGCTTGGCCTCAGCCTAGAAGCTTTCGACAGGGGTTCATCAGAAGCCACCACATGGGACGGCACCACTGAGGGCGTGGAACACCGTGCAGCGGCGAACGCGATTAAGGAAGCAAACTGCCCAATACACAAGGTTACCTATTACCTGGCCAAACCGACGTTCGCCATCAGGCAGTCCAAAAACCTTCCCCCAGCCAACTACGCAAAGAAAAATGTGCCGTCACAATACAAATGGTGCGCGTTCGACGCGTTTGACGGATTATACGACCCGACCTGCTTAGCCTCAGAGCTGCCCTATGACGCCCCCTCTGAAATAGACCGGATGGCATATGCCACTTTCAAAACCATACAGATCAAGACCGCTAATGATCAAAAGGGGTTCAACCTCAACTACAACCCCAACGTCACTCAAGCACGGCTCCCAAACACACCTCTGCCCGCCCTCCCTGAACCAGCATCAGACCTGCATCATCCTGTTCATACTAATCCTAGGCACAGTTTATAATTTACTTCAACAGCCTCAACCTCCACCATGTGAAATAACCATAAATGGGCACACCATATCCATCAAAGGCAACTGCTATCATACTACCTCTAGCTAGGATTTGTTAGGTTTCCCACTCTTGTGAATTAAACATTAAAGATGGAACGAGCTATACAAGGAAACGACGCTAGGGAACAAGCTAACAGTGAACGTTGGGATGGAGGATCAGGAGGTACCACTTCTCCCTTCAAACTTCCTGACGAAAGTCCGAGTTGGACTGAGTGGCGGCTACATAACGATGAGACGAATTCGAATCAAGATAATCCCCTTGGTTTCAAGGAAAGCTGGGGTTTCGGGAAAGTTGTATTTAAGAGATATCTCAGATACGACAGGACGGAAGCTTCACTGCACAGAGTCCTTGGATCTTGGACGGGAGATTCGGTTAACTATGCAGCATCTCGATTTTTCGGTTTCGACCAGATCGGATGTACCTATAGTATTCGGTTTCGAGGAGTTAGTATCACCGTTTCTGGAGGGTCGCGAACTCTTCAGCATCTCTGTGAGATGGCAATTCGGTCTAAGCAAGAACTGCTACAGCTTGCCCCAATCGAAGTGGAAAGTAATGTATCAAGAGGATGCCCTGAAGGTACTGAGACCTTCGAAAAAGAAAGCGAGggcagtggagagggcagaggaagtctgctaacatgcggtgacgtcgaggagaatcctggcccaatggactataaggaccacgacggagactacaaggatcatgatattgattacaaagacgatgacgataagatggctcctaagaagaagcggaaggttggtattcacggggtgcctgcggctATGATCAAGGTGTACAGGTACGAGATCGTGAAGCCGCTCGACCTCGACTGGAAGGAGTTCGGCACCATCCTCAGGCAGCTCCAGCAGGAGACAAGGTTCGCCCTCAACAAGGCCACCCAGCTCGCCTGGGAGTGGATGGGCTTCTCCTCCGACTACAAGGACAACCACGGCGAGTACCCGAAGTCCAAGGACATCCTCGGCTACACCAACGTGCACGGCTACGCCTACCACACCATCAAGACCAAGGCCTACAGGCTCAACTCCGGCAACCTCTCCCAGACCATCAAGAGGGCCACCGACAGGTTCAAGGCCTACCAGAAGGAGATCCTCAGGGGCGACATGTCCATCCCGTCCTACAAGAGGGACCACCCGCTCGACCTCATCAAGGAGAACATCTCCGTGAACAGGATGAACCACGGCGACTACATCGCCTCCCTCTCCCTCCTCTCCAACCCGGCCAAGCAGGAGATGAACGTGAAGAGGAAGATCTCCGTGATCATCATCGTGAGGGGCGCCGGCAAGACCATCATGGACAGGATCCTCTCCGGCGAGTACCAGGTGTCCGCCTCCCAGATCATCCACAAGGACAGGAAGAACAAGTGGTACCTCAACATCTCCTACAGGTTCGAGCCGCAGACCAGGGTGCTCGACCTCAACAAGATCATGGGCATCGACCTCGGCGTGGCCGTGGCCGCGTACATGGCCTTCCAGCACACCCCGGCCAGGTACAAGCTCGAGGGCGGCGAGATCGAGAACTTCAGGAGGCAGGTGGAGTCCAGGAGGATCTCCATGCTCAGGCAGGGCAAGTACGCCGGCGGCGCCAGGGGCGGCCACGGCAGGGACAAGAGGATCAAGCCGATCGAGCAGCTCAGGGACAAGATCGCCAACTTCAGGGACACCACCAACCACAGGTACTCCAGGTACATCGTGGACATGGCCATCAAGGAGGGCTGCGGCACCATCCAGATGGAGGACCTCACCAACATCAGGGACATCGGCTCCAGGTTCCTCCAGAACTGGACCTACTACGACCTCCAGCAGAAGATCATCTACAAGGCCGAGGAGGCCGGCATCAAGGTGATCAAGATCGACCCGCAGTACACCTCCCAGAGGTGCTCCGAGTGCGGCAACATCGACTCCGGCAACAGGATCGGCCAGGCCATCTTCAAGTGCAGGGCCTGCGGCTACGAGGCCAACGCCGACTACAACGCCGCCAGGAACATCGCCATCCCGAACATCGACAAGATCATCGCCGAGTCCATCAAGaagcgtcctgctgccaccaaaaaggccggacaggctaagaaaaagaagtagAACAAAGCACCAGTGGTCTAGTGGTAGAATAGTACCCTGCCACGGTACAGACCCGGGTTCGATTCCCGGCTGGTGCATCGTCGGTTCAGCGACGATAAGCCGAGAAGTGCCAATAAAACTGTTAAGTGGTTTGGTAACGCTCGGTAAGGTCCGAAAGGAGAACCACTGAACNNNNNNNNNNNNNNNNNNNNAACAAAGCACCAGTGGTCTAGTGGTAGAATAGTACCCTGCCACGGTACAGACCCGGGTTCGATTCCCGGCTGGTGCAACGTTGCATGATCGTAAAACATGCCCGGCTTACCGTGAGCCGCCTTTGAAAGAAAGGTTTACACGGATCCTGTTGTGTCACGCACGTACCTAACTTGTGTCAGCAGAATAAAGACCTTTTGGTTTCTACAGTTTTTTCCA | CP-CPpro-p19-T2A-3xFlag-SV40NLS-AsCas12f1-HKRA-NLS-tRNA-sgRNA-tRNA-3’UTR |
| AsCas12f1_HKRA_-eGFP | ATGGAACGAGCTATACAAGGAAACGACGCTAGGGAACAAGCTAACAGTGAACGTTGGGATGGAGGATCAGGAGGTACCACTTCTCCCTTCAAACTTCCTGACGAAAGTCCGAGTTGGACTGAGTGGCGGCTACATAACGATGAGACGAATTCGAATCAAGATAATCCCCTTGGTTTCAAGGAAAGCTGGGGTTTCGGGAAAGTTGTATTTAAGAGATATCTCAGATACGACAGGACGGAAGCTTCACTGCACAGAGTCCTTGGATCTTGGACGGGAGATTCGGTTAACTATGCAGCATCTCGATTTTTCGGTTTCGACCAGATCGGATGTACCTATAGTATTCGGTTTCGAGGAGTTAGTATCACCGTTTCTGGAGGGTCGCGAACTCTTCAGCATCTCTGTGAGATGGCAATTCGGTCTAAGCAAGAACTGCTACAGCTTGCCCCAATCGAAGTGGAAAGTAATGTATCAAGAGGATGCCCTGAAGGTACTGAGACCTTCGAAAAAGAAAGCGAGggcagtggagagggcagaggaagtctgctaacatgcggtgacgtcgaggagaatcctggcccaatggactataaggaccacgacggagactacaaggatcatgatattgattacaaagacgatgacgataagatggctcctaagaagaagcggaaggttggtattcacggggtgcctgcggctATGATCAAGGTGTACAGGTACGAGATCGTGAAGCCGCTCGACCTCGACTGGAAGGAGTTCGGCACCATCCTCAGGCAGCTCCAGCAGGAGACAAGGTTCGCCCTCAACAAGGCCACCCAGCTCGCCTGGGAGTGGATGGGCTTCTCCTCCGACTACAAGGACAACCACGGCGAGTACCCGAAGTCCAAGGACATCCTCGGCTACACCAACGTGCACGGCTACGCCTACCACACCATCAAGACCAAGGCCTACAGGCTCAACTCCGGCAACCTCTCCCAGACCATCAAGAGGGCCACCGACAGGTTCAAGGCCTACCAGAAGGAGATCCTCAGGGGCGACATGTCCATCCCGTCCTACAAGAGGGACCACCCGCTCGACCTCATCAAGGAGAACATCTCCGTGAACAGGATGAACCACGGCGACTACATCGCCTCCCTCTCCCTCCTCTCCAACCCGGCCAAGCAGGAGATGAACGTGAAGAGGAAGATCTCCGTGATCATCATCGTGAGGGGCGCCGGCAAGACCATCATGGACAGGATCCTCTCCGGCGAGTACCAGGTGTCCGCCTCCCAGATCATCCACAAGGACAGGAAGAACAAGTGGTACCTCAACATCTCCTACAGGTTCGAGCCGCAGACCAGGGTGCTCGACCTCAACAAGATCATGGGCATCGACCTCGGCGTGGCCGTGGCCGCGTACATGGCCTTCCAGCACACCCCGGCCAGGTACAAGCTCGAGGGCGGCGAGATCGAGAACTTCAGGAGGCAGGTGGAGTCCAGGAGGATCTCCATGCTCAGGCAGGGCAAGTACGCCGGCGGCGCCAGGGGCGGCCACGGCAGGGACAAGAGGATCAAGCCGATCGAGCAGCTCAGGGACAAGATCGCCAACTTCAGGGACACCACCAACCACAGGTACTCCAGGTACATCGTGGACATGGCCATCAAGGAGGGCTGCGGCACCATCCAGATGGAGGACCTCACCAACATCAGGGACATCGGCTCCAGGTTCCTCCAGAACTGGACCTACTACGACCTCCAGCAGAAGATCATCTACAAGGCCGAGGAGGCCGGCATCAAGGTGATCAAGATCGACCCGCAGTACACCTCCCAGAGGTGCTCCGAGTGCGGCAACATCGACTCCGGCAACAGGATCGGCCAGGCCATCTTCAAGTGCAGGGCCTGCGGCTACGAGGCCAACGCCGACTACAACGCCGCCAGGAACATCGCCATCCCGAACATCGACAAGATCATCGCCGAGTCCATCAAG GGTGGCGGTGGCTCGGGCGGAGGTGGGTCGGGTGGCGGCGGATCAgtgagcaagggcgaggagctgttcaccggggtggtgcccatcctggtcgagctggacggcgacgtaaacggccacaagttcagcgtgtccggcgagggcgagggcgatgccacctacggcaagctgaccctgaagttcatctgcaccaccggcaagctgcccgtgccctggcccaccctcgtgaccaccttcacctacggcgtgcagtgcttcagccgctaccccgaccacatgaagcagcacgacttcttcaagtccgccatgcccgaaggctacgtccaggagcgcaccatcttcttcaaggacgacggcaactacaagacccgcgccgaggtgaagttcgagggcgacaccctggtgaaccgcatcgagctgaagggcatcgacttcaaggaggacggcaacatcctggggcacaagctggagtacaactacaacagccacaacgtctatatcatggccgacaagcagaagaacggcatcaaggtgaacttcaagatccgccacaacatcgaggacggcagcgtgcagctcgccgaccactaccagcagaacacccccatcggcgacggccccgtgctgctgcccgacaaccactacctgagcacccagtccgccctgagcaaagaccccaacgagaagcgcgatcacatggtcctgctggagttcgtgaccgccgccgggatcactcacggcatggacgagctgtacaagaagcgtcctgctgccaccaaaaaggccggacaggctaagaaaaagaagtag | p19-T2A-3xFlag-SV40NLS-AsCas12f1-HKRA-(G4S)3-eGFP-NLS |

**Supplemental Table 3.** The List of primers used in this study.

| Primer | Sequence (5' → 3') | Application |
| --- | --- | --- |
| **Primers used for RT-PCR** | | |
| Ba32 | CCAAAAGGTCTTTATTCTGC | BaMV 3’-UTR specific primer |
| ORF5-jd-F | GTCTGATGCTGGTTCAGGGAGGGCG | For RT-PCR (BaMV ORF5) |
| ORF5-jd-R | ATGTCCGGAACTGGTACAGGTACAGGG |  |
| Cas9-F | ATCACCACCAAGCTGGGAAAGGTCGATAC | For RT-PCR (SpCas9) |
| Cas9-R | GACAAGAAGTACTCCATCGGCCTCGACATC |  |
| Cas9-sgRNA-F | GCACCGACTCGGTGCCAC | For RT-PCR (sgRNA of SpCas9) |
| Cas9-sgRNA-R | GTTTTAGAGCTAGAAATAGC |  |
| NbActin1-F | TGAAGATCCTCACAGAGCGTGG | For RT-PCR (Gene ID AY594294) |
| NbActin1-R | TTGTATGTGGTCTCGTGGATTC |  |
| AsCas12f1-F | CTTGATGGACTCGGCGATGATC | For RT-PCR (AsCas12f1) |
| AsCas12f1-R | ATGATCAAGGTGTACAGGTAC |  |
| enCas12j8-F | CACGGCCTCTCTCAGCACGAC | For RT-PCR (enCas12j8) |
| enCas12j8-R | ATCAAGCCAACCGTCTCCCAG |  |
| vCas12j2-F | AGTTTGTGATGGCTCTTGAG | For RT-PCR (vCas12j2) |
| vCas12j2-R | ATGCCAAAGCCTGCTGTTGAG |  |
| nCas12j2-F | CGACGTCTGAGAAGGCTCCTG | For RT-PCR (nCas12j2) |
| nCas12j2-R | ATGCCAAAGCCAGCCGTCGAG |  |
| Cas12f1-sgRNA-F | GTTCAGTGGTTCTCCTTTCG | For RT-PCR (sgRNA of AsCas12f1) |
| Cas12f1-sgRNA-R | TCGTCGGTTCAGCGACGATA |  |
| PheActin-F | TACGCTTCCTCACGCTAT | For RT-PCR (Gene ID PH02Gene38192) |
| PheActin-R | GCTTCTCCTTTATGTCCCT |  |
| **Primers used for Hi-TOM** | | |
| HiTom-gPhePDS1-F | ggagtgagtacggtgtgcCCAAGCACTGAAAAGTAG | 258-bp PCR product of PhePDS1 for Hi-TOM |
| HiTom-gPhePDS1-R | gagttggatgctggatggTTCTAAAAAATGAAGAGAG |  |
| HiTom-gPhePDS2-F | ggagtgagtacggtgtgcTCCACTGTTATCATTATTTG | 190-bp PCR product of PhePDS2 for Hi-TOM |
| HiTom-gPhePDS2-R | gagttggatgctggatggTATACGCTGCTAATGGAAAC |  |
| HiTOM-NbPDS-F | ggagtgagtacggtgtgcGGGAACTGAAAGTCAAGATG | 168-bp PCR product of NbPDS for Hi-TOM |
| HiTOM-NbPDS-R | gagttggatgctggatggAGCATCACACTTTCGCATTC |  |
| HiTOM-NbPSY-F | ggagtgagtacggtgtgcCTTTAGTGAAGAGGCAGTTG | 156-bp PCR product of NbPSY for Hi-TOM |
| HiTOM-NbPSY-R | gagttggatgctggatggAAAGAAGCTAACCTAAGTAA |  |
| HiTOM-NbRDR6-F | ggagtgagtacggtgtgcCCAAAGGGTGAAAAGTATTC | 172-bp PCR product of NbRDR6 for Hi-TOM |
| HiTOM-NbRDR6-R | gagttggatgctggatggCTGTTACTGAACCTCCCCATC |  |
| HiTom-PheRDR6-F | ggagtgagtacggtgtgcTCTTCCACCAGAAGTGGAGC | 132-bp PCR product of PheRDR6 for Hi-TOM |
| HiTom-PheRDR6-R | gagttggatgctggatggCATAAAAGTTCAGCACATTG |  |
| **Primers used for PCR-RE** | | |
| NbPSY-F | ATGTCTGTTGCCTTGTTATGG | 593-bp PCR product of NbPSY-A for PCR-RE |
| NbPSY-R | AGAAACCTCACCATATATTG |  |
| NbPDS-F | AACTTTACTCATAGTGCGAG | 482-bp PCR product of NbPDS-B for PCR-RE |
| NbPDS-R | GTGATCATAAATTCAGCATC |  |
| NbRDR6-F | ATTGTAGAGGTTAGGAGATTGGTTAT | 460-bp PCR product of NbRDR6-B for PCR-RE |
| NbRDR6-R | GCATAAGTTGATGAAAAGCACTGTCC |  |
| gPhePDS1-F | ATGGATACTGGCTGCCTGTC | 616-bp PCR product of PhePDS-B for PCR-RE |
| gPhePDS1-R | TTGAGATGCGATAAGCAAGC |  |
| gPhePDS2-F | GCTTGCTTATCGCATCTCAA | 790-bp PCR product of PhePDS-B for PCR-RE |
| gPhePDS2-R | AAGGGCGCTGGCAAAGTTTC |  |
| gPheRDR6-F | GGAACCCCAAGCTTCTTCAC | 538-bp PCR product of PheRDR6 for PCR-RE |
| gPheRDR6-R | CTCTCATTGACCTCATCAGG |  |
| **Primers used for cloning BaMV plasmids** | | |
| STU-Cas9-F | GCTATTTCTAGCTCTAAAACtctagaCTACTTCTTTTTCTTAGCCTGTC | Used for cloning BaMV-STU-Cas9 |
| STU-Cas9-R | GACGTCGAGGAGAATCCTGGCCCAATGGACTACAAAGACCATGAC |  |
| gRNA scaffold-F | GTATGAACAGGATGATGCAGAAAAAAAGCACCGACTCGGTGCC |  |
| gRNA scaffold-R | GACAGGCTAAGAAAAAGAAGTAGtctagaGTTTTAGAGCTAGAAATAG |  |
| STU-Cas9-tRNA-F | TAGACCACTGGTGCTTTGTTCTACTTCTTTTTCTTAGCCTGTC | Used for cloning BaMV-STU-Cas9-tRNA |
| STU-Cas9-tRNA-R | GACGTCGAGGAGAATCCTGGCCCAATGGACTACAAAGACCATGAC |  |
| preTRNA-F1 | GTATGAACAGGATGATGCAGTGCACCAGCCGGGAATCGAAC |  |
| preTRNA-R1 | AAGTGGCACCGAGTCGGTGCAACAAAGCACCAGTGGTCTAG |  |
| preTRNA-F2 | GCTATTTCTAGCTCTAAAACtctagaTGCACCAGCCGGGAATCGAAC |  |
| preTRNA-F2 | AGGCCGGACAGGCTAAGAAAAAGAAGTAGAACAAAGCACCAGTGGTCTAG |  |
| gRNA scaffold-tRNA-F | CTAGACCACTGGTGCTTTGTTGCACCGACTCGGTGCCACTT |  |
| gRNA scaffold-tRNA-R | TTCGATTCCCGGCTGGTGCAtctagaGTTTTAGAGCTAGAAATAGC |  |
| enCas12j8-F | AGTATGAACAGGATGATGCAGtctagaTCACTTCTTCTTCTTGGCTTGAC | Used for cloning BaMV-STU-enCas12j8-tRNA |
| enCas12j8-R | ACAAAGACGATGACGATAAGATGGCCCCAAAGAAGAAGCG |  |
| 12j8 crRNA-F | GTATGAACAGGATGATGCAGtctagaGTCTCCTCGTAAGGAGCAATC |  |
| 12j8 crRNA-R | GTCAAGCCAAGAAGAAGAAGtgaAACAAAGCACCAGTGGTCTAG |  |
| tRNA-F | GTATGAACAGGATGATGCAGTGCACCAGCCGGGAATCG |  |
| tRNA-R | ATTGCTCCTTACGAGGAGACtctagaAACAAAGCACCAGTGGTC |  |
| vCas12j2-F | CTAGACCACTGGTGCTTTGTTCTACTTCTTCTTCTTGGCTTGAC | Used for cloning BaMV-STU-vCas12j2-tRNA and BaMV-STU-nCas12j2-tRNA |
| vCas12j2-R | CAAAGAAGAAGCGTAAGGTCATGCCAAAGCCTGCTGTTGAG |  |
| nCas12j2-F | CTAGACCACTGGTGCTTTGTTCTACTTCTTCTTCTTGGCTTGAC |  |
| nCas12j2-R | TTCACGGGGTGCCTGCGGCTATGCCAAAGCCAGCCGTCGAG |  |
| tRNA-F | GTATGAACAGGATGATGCAGTGCACCAGCCGGGAATCGAAC |  |
| tRNA-R | GTCAAGCCAAGAAGAAGAAGTAGAACAAAGCACCAGTGGTCTAG |  |
| 12j2 crRNA-F | GTCCCCTCGTaAGGGGCAATCGTTGTGCACCAGCCGGGAATCG |  |
| 12j2 crRNA-R | CAACGATTGCCCCTtACGAGGGGACtctagaAACAAAGCACCAGTGGTCTAG |  |
| HKRA-F | AGTATGAACAGGATGATGCAGCTACTTCTTTTTCTTAGCCTGTC | Used for cloning BaMV-STU-AsCas12f1_HKRA_-tRNA and BaMV-STU-AsCas12f1_YHAM_-tRNA |
| HKRA-R | GATTACAAAGACGATGACGATAAGATGGCTCCTAAGAAGAAGCGG |  |
| YHAM-F | AGTATGAACAGGATGATGCAGCTACTTCTTTTTCTTAGCCTGTC |  |
| YHAM-R | GATTACAAAGACGATGACGATAAGATGGCTCCTAAGAAGAAGCGG |  |
| 12f1 crRNA-F | TAGACCACTGGTGCTTTGTTtctagaGTTCAGTGGTTCTCCTTTCG |  |
| 12f1 crRNA-R | TTCGATTCCCGGCTGGTGCATCGTCGGTTCAGCGACGATAAG |  |
| tRNA-F | GTATGAACAGGATGATGCAGTGCACCAGCCGGGAATCG |  |
| tRNA-R | AGGCCGGACAGGCTAAGAAAAAGAAGTAGAACAAAGCACCAGTGGTCTAG |  |
| CP1-HKRA-F | GTTTTACGATCATGCAACGTTGCACCAGCCGGGAATCGAAC | Used for cloning BaMV-STU-AsCas12f1_HKRA_-tRNA-CP1 |
| CP1-HKRA-R | TCCCTGAACCAGCATCAGACGGCAGTGGAGAGGGCAGAGG |  |
| CP2-HKRA-F | GTTTTACGATCATGCAACGTTGCACCAGCCGGGAATCGAAC | Used for cloning BaMV-STU-AsCas12f1_HKRA_-tRNA-CP2 |
| CP2-HKRA-R | CTGAACCAGCATCAGACTAACTGCATCATCCTGTTCATAC |  |
| HKRA_eGFP-F | TTTGGTGGCAGCAGGACGCTTCTTGTACAGCTCGTCCATGC | Used for cloning pBaMV-AsCas12f1_HKRA_-eGFP, pBaMV-AsCas12f1_HKRA_-eGFP-CP1 and pBaMV-AsCas12f1_HKRA_-eGFP-CP2 |
| HKRA_eGFP-R | CATCGCCGAGTCCATCAAGGGTGGCGGTGGCTCGGGCGG |  |
| **Primers used for used for sgRNA Cloning** | | |
| Cas9-gNbPDS-F | GCTATTTCTAGCTCTAAAACCATGGAGTCGCTACTACCAATGCACCAGCCGGGAATCGAA | 20-bp Cas9 gNbPDS sequences |
| Cas9-gNbPDS-R | TTCGATTCCCGGCTGGTGCATTGGTAGTAGCGACTCCATGGTTTTAGAGCTAGAAATAGC |  |
| Cas12f-gNbPDS-F | CTAGACCACTGGTGCTTTGTTCCCCATGGAGTCGCTACTACGTTCAGTGGTTCTCCTTTCG | 20-bp AsCas12f1 gNbPDS sequences |
| Cas12f-gNbPDS-R | CGAAAGGAGAACCACTGAACGTAGTAGCGACTCCATGGGGAACAAAGCACCAGTGGTCTAG |  |
| Cas12j8-gNbPDS-F | CTAGACCACTGGTGCTTTGTTCCATGGAGTCGCTACTACGTCTCCTCGTAAGGAGCAAT | 18-bp enCas12j8 gNbPDS sequences |
| Cas12j8-gNbPDS-R | ATTGCTCCTTACGAGGAGACGTAGTAGCGACTCCATGGAACAAAGCACCAGTGGTCTAG |  |
| Cas12j2-gNbPDS-F | CTAGACCACTGGTGCTTTGTTCCATGGAGTCGCTACTACGTCCCCTCGTaAGGGGCAATCGTTG | 18-bp Cas12j2 gNbPDS sequences |
| Cas12j2-gNbPDS-R | CAACGATTGCCCCTtACGAGGGGACGTAGTAGCGACTCCATGGAACAAAGCACCAGTGGTCTAG |  |
| Cas12f-gNbPSY-F | CTAGACCACTGGTGCTTTGTTTATGCTTCACTCAACAAGCTGTTCAGTGGTTCTCCTTTCG | 20-bp AsCas12f1 gNbPSY sequences |
| Cas12f-gNbPSY-R | CGAAAGGAGAACCACTGAACAGCTTGTTGAGTGAAGCATAAACAAAGCACCAGTGGTCTAG |  |
| Cas12f-gNbRDR6-F | TAGACCACTGGTGCTTTGTTAGCTGGTTAGCTGAGAATGCGTTCAGTGGTTCTCCTTTCG | 20-bp AsCas12f1 gNbRDR6 sequences |
| Cas12f-gNbRDR6-R | CGAAAGGAGAACCACTGAACGCATTCTCAGCTAACCAGCTAACAAAGCACCAGTGGTCTA |  |
| Cas12f-gPhePDS1-F | CTAGACCACTGGTGCTTTGTTAGAGCACCAAGTCCACGGCGGTTCAGTGGTTCTCCTTTCG | 20-bp AsCas12f1 gPhePDS1 sequences |
| Cas12f-gPhePDS1-R | CGAAAGGAGAACCACTGAACCGCCGTGGACTTGGTGCTCTAACAAAGCACCAGTGGTCTAG |  |
| Cas12f-gPhePDS2-F | CTAGACCACTGGTGCTTTGTTTATGAAGCCCGGTCTCATACGTTCAGTGGTTCTCCTTTCG | 20-bp AsCas12f1 gPhePDS2 sequences |
| Cas12f-gPhePDS2-R | CGAAAGGAGAACCACTGAACGTATGAGACCGGGCTTCATAAACAAAGCACCAGTGGTCTAG |  |
| Note: gRNA sequences highlighted in green | | |
