# Supplementary material for: BaMV‐Vectored Compact AsCas12f1‐HKRA Enables Transgene‐Free Genome Editing in Moso Bamboo ( Phyllostachys edulis )
Source: Plant Biotechnol J. 2025 Dec 2;24(4):2220–2. doi: 10.1111/pbi.70474 (PMC13140604; doi:10.1111/pbi.70474)
Supplement: Supplementary file 3 — Appendix S3: Figures S1–S5 (details provided in Appendix S3). Figure S1: BaMV‐mediated single transcript CRISPR‐Cas9 system. Figure S2: BaMV‐mediated single transcript CRISPR‐Cas12 system. Figure S3: Multiplexed endogenous genome editing. Figure S4: Evaluation of AsCas12f1HKRA‐tgtRNA insertions at distinct sites. Figure S5: Genome editing in Phyllostachys edulis. [file PBI-24-2220-s003.zip › pbi70474-sup-0003-AppendixS3/V12-Appendix S3 Figure-legend.docx]

**Figure legends**

**Figure 1.** **BaMV-mediated single transcript CRISPR-Cas system in genome editing.**

(a) Schematic diagrams of the BaMV-Cas*^STU^* systems. The red curved arrow represents the BaMV ORF5 promoter, and the red asterisk represents the stop codon. (b) Histogram presented the editing efficiencies induced by three Cas9 systems at *NbPDS* target sites by amplicon deep sequencing. gRNA-Cas9: the BaMV-gRNA-Cas9 system, STU-Cas9: the BaMV-STU-Cas9 system, STU-Cas9-tRNA: the BaMV-STU-Cas9-tRNA system. Each point represents a biological replication from an independent experiment (n>3). Data are presented as the mean values ± SD. ANOVA: ns: *P* > 0.05, *: *P* < 0.05, **: *P* < 0.01. (c) Phenotypes of *N. benthamiana* plants infected with the BaMV-STU-AsCas12f1_HKRA_-tRNA system targeting *NbPDS*. Scale bars: 1 cm. (d) Histogram presented the editing efficiencies induced by the BaMV-Cas12*^STU^* systems at *NbPDS* target sites. Each point represents a biological replication from an independent experiment (n>3). Data are presented as the mean values ± SD. ANOVA: ns: *P* > 0.05, *: *P* < 0.05, **: *P* < 0.01. (e) PCR-RE detection and Sanger sequencing of *NbPDS* and *NbPSY* genes mediated by HKRA_tg1tg2t in systemic leaves. HKRA_tg1tg2t: the BaMV-STU-AsCas12f1_HKRA_-tRNA system dual-targeting *NbPSY* and *NbPDS*. Red arrows indicate digestion-resistant fragments. (f) Histogram presented the editing efficiencies of *NbPDS,* *NbPSY* and *NbRDR6* genes mediated by HKRA_tg1tg2t and HKRA_tg1tg2tg3t. HKRA_tg1t: the BaMV-STU-AsCas12f1_HKRA_-tRNA system targeting *NbPDS,* HKRA_tg1tg2tg3t: the BaMV-STU-AsCas12f1_HKRA_-tRNA system triple-targeting *NbPDS*, *NbPSY* and *NbRDR6*. Each point represents a biological replication from an independent experiment (n>3). Data are presented as the mean values ± SD. ANOVA: ns: *P* > 0.05, *: *P* < 0.05, **: *P* < 0.01. (g) Histogram presented the editing efficiencies induced by the BaMV-Cas*^STU^* systems at *PheRDR6* target sites. Each point represents a biological replication from an independent experiment (n>3). Data are presented as the mean values ± SD. ANOVA: ns: *P* > 0.05, *: *P* < 0.05, **: *P* < 0.01. (h) Phenotype of *P. edulis* infected with BaMV-STU-AsCas12f1_HKRA_-tRNA system targeting the gPhePDS-1 site. Scale bars: 1 cm. (i) T7EI detection of gPhePDS1 target sites in newly emerged tiller buds and systemic leaves. Red arrows indicate restricted products. (j) The editing efficiencies of *PhePDS* at gPhePDS1 and gPhePDS2 target sites were determined by amplicon deep sequencing. Each point represents a biological replication from an independent experiment (n>3). Data are presented as the mean values ± SD. ANOVA: ns: *P* > 0.05, *: *P* < 0.05, **: *P* < 0.01. (k) Mutation patterns and frequencies of *PhePDS* at gPhePDS1 target sites were detected by deep sequencing.

**Figure S1. BaMV-mediated single transcript CRISPR-Cas9 system in *N. benthamiana* genome editing.**

(a) Phenotype of *N. benthamiana* plants at 4 weeks after agro-infiltration. Mock: mock-infection, BaMV: BaMV infection. BaMV-Cas9: the BaMV-gRNA-Cas9 system. (b) RT-PCR and western blot detection of gRNA and Cas9 in system leaves with BaMV derivatives after 5 weeks. (c) PCR-RE detection of *NbPDS* induced by three Cas9 systems within symptomatic systemic leaves of *N. benthamiana*. M: DNA marker. Mock/U and Mock/D denote the PCR products from mock-infected plants without or with restriction digestion, respectively. Cas9: the BaMV-gRNA-Cas9 system, STU-Cas9: the BaMV-STU-Cas9 system, STU-Cas9-tRNA: the BaMV-STU-Cas9-tRNA system. Red arrows indicate digestion-resistant fragments. (d) Deletion patterns at the *NbPDS* target site, displaying with their respective positions, lengths, and corresponding frequencies. (e) RT-PCR detection of the transcriptional level of gRNA in *N. benthamiana* systemic leaves.

**Figure S2. BaMV-mediated single transcript CRISPR-Cas12 system in *N. benthamiana* genome editing.**

(a) Vector architectures of the BaMV-Cas12*^STU^* systems and domain organization and dsDNA cleavage scheme of AsCas12f and Cas12j. HNH, REC, and RuvC domains are indicated. Protein lengths are drawn to scale. aa: amino acid, TS: DNA target strand; NTS: DNA non-target stand. (b) Phenotypes of *N. benthamiana* plants at 4 weeks after agro-infiltration. (c) Western blot and RT-PCR detection of Cas12 proteins and Cas12 transcripts in tobacco system leaves with BaMV derivatives after 5 weeks. (d) PCR-RE detection of *NbPDS* induced by the BaMV-Cas12*^STU^* systems within symptomatic systemic leaves of *N. benthamiana*. M: DNA marker. Mock/U and Mock/D denote the PCR products from mock-infected plants without or with restriction digestion, respectively. STU-YHAM-tRNA: the BaMV-STU-AsCas12f1_YHAM_-tRNA system, STU-vCas12j2-tRNA: the BaMV-STU-vCas12j2-tRNA system, STU-nCas12j2-tRNA: the BaMV-STU-nCas12j2-tRNA system. Red arrows indicate digestion-resistant fragments. (e) Deletion patterns induced by the BaMV-mediated CRISPR-AsCas12f1 system and CRISPR-Cas12j8 system at the *NbPDS* target site, displaying with their respective positions, lengths, and corresponding frequencies. (f) Phenotypic screening of T1 progeny from gene-edited tobacco on medium. Scale bars: 1 cm.

**Figure S3. Multiplexed endogenous genome editing in *N. benthamiana* using BaMV-STU-AsCas12f1_HKRA_-tRNA.**

(a) Schematic diagrams of BaMV-mediated AsCas12f1_HKRA_ multiplex editing design and the phenotype of *N. benthamiana* infected with the multiplex editing vectors. BaMV-STU-AsCas12f1_HKRA_-tRNA: target *NbPDS*, HKRA_tg1tg2t: dual-targeting *NbPSY* and *NbPDS*, HKRA_tg1tg2tg3t: triple-targeting *NbPDS, NbPSY* and *NbRDR6*. Scale bars: 1 cm. (b) PCR-RE detection and Sanger sequencing of *NbPDS, NbPSY* and *NbRDR6* genes mediated by HKRA_tg1tg2tg3t in systemic leaves. M: DNA marker. Mock/U and Mock/D denote the PCR products from mock-infected plants without or with restriction digestion, respectively. Red arrows indicate digestion-resistant fragments. (c) Mutation patterns and frequencies at *NbPDS, NbPSY* and *NbRDR6*, showing respective positions, lengths, and corresponding frequencies.

**Figure S4. Functional evaluation of AsCas12f1_HKRA_-tgtRNA insertions at distinct BaMV genomic sites.**

(a) Schematic diagram of plasmid carrying AsCas12f1_HKRA_-eGFP at different insertion sites in BaMV and inserting AsCas12f1_HKRA_-tgtRNA at two different sites after BaMV ORF5. HKRA_eGFP: pBaMV-2-AsCas12f1_HKRA_-eGFP, HKRA_eGFP_CP1: pBaMV-3-CP-2A-AsCas12f1_HKRA_-eGFP, HKRA_eGFP_CP2: pBaMV-3-CP-ORF5promoter-AsCas12f1_HKRA_-eGFP, HKRA-CP1: BaMV-STU-AsCas12f1_HKRA_-tRNA-CP1, HKRA-CP2: BaMV-STU-AsCas12f1_HKRA_-tRNA-CP2. (b) GFP fluorescence in *N. benthamiana* 3 weeks post-infection with distinct BaMV-derived clones. White arrow indicates fluorescence signal locations. Scale bars: 1 cm. (c) Phenotype of *N. benthamiana* infected with BaMV carrying AsCas12f1_HKRA_-tgtRNA elements. Scale bars: 1 cm. (d) Western blot detection of AsCas12f1_HKRA_ in inoculated leaves, systemic leaves, and stems of *N. benthamiana*. (e) PCR-RE detection of *NbPDS* in tobacco systemic leaves, and stems and inoculated leaves. (f) Histogram presented editing efficiencies at target gene loci in *NbPDS*. Each point represents a biological replication from an independent experiment (n = 6). ANOVA: ns: *P* > 0.05, *: *P* < 0.05, **: *P* < 0.01.

**Figure S5. Genome editing in *Phyllostachys edulis* using BaMV-STU-AsCas12f1_HKRA_-tRNA system.**

(a) Diagrams of the BaMV-gRNA-Cas9, BaMV-STU-Cas9, BaMV-STU-Cas9-tRNA and BaMV-STU-AsCas12f1_HKRA_-tRNA systems for *PheRDR6* and *PhePDS* targeting. (b) T7EI detection of *PheRDR6* target site in symptomatic leaves. (c) Phenotype of *P. edulis* infected with BaMV-STU-AsCas12f1_HKRA_-tRNA system targeting *PhePDS*. Mock: mock-infection, Scale bars: 1 cm. (d) T7EI detection of gPhePDS2 target sites in newly emerged tiller buds and systemic leaves. (e) Mutation patterns and frequencies of *PhePDS* at gPhePDS2 target sites were detected by deep sequencing.
