# Supplementary figures and images for: BaMV‐Vectored Compact AsCas12f1‐HKRA Enables Transgene‐Free Genome Editing in Moso Bamboo ( Phyllostachys edulis )
Source: Plant Biotechnol J. 2025 Dec 2;24(4):2220–2. doi: 10.1111/pbi.70474 (PMC13140604; doi:10.1111/pbi.70474)

Figure S1

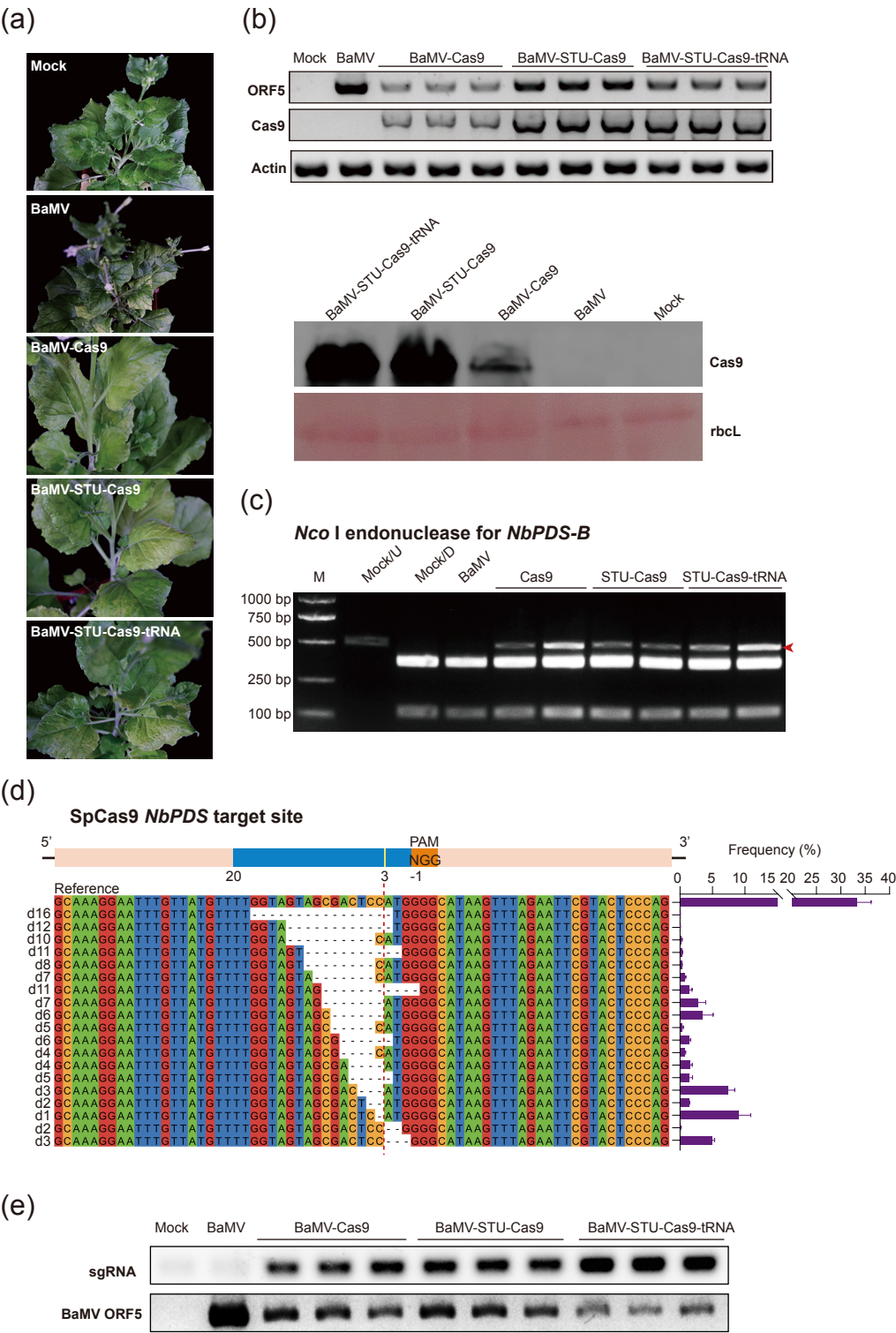

**Figure S2**

(a)

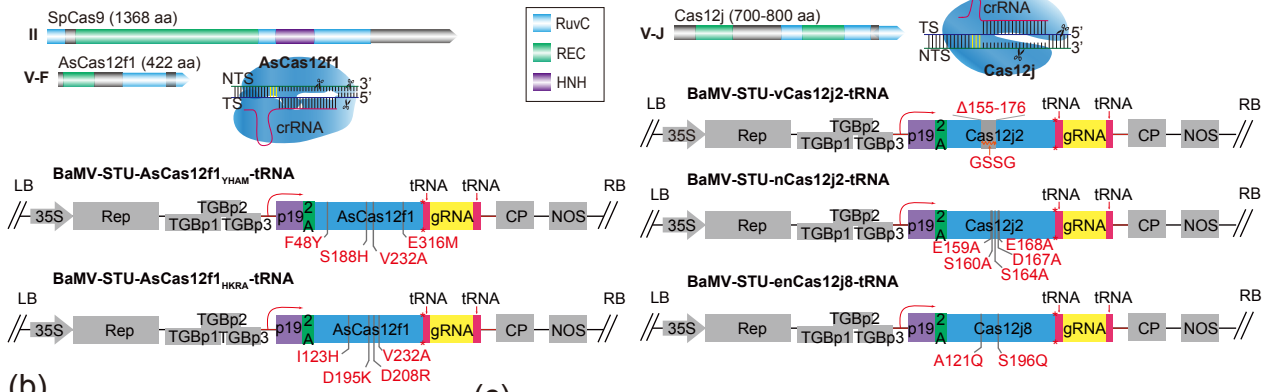

(b)

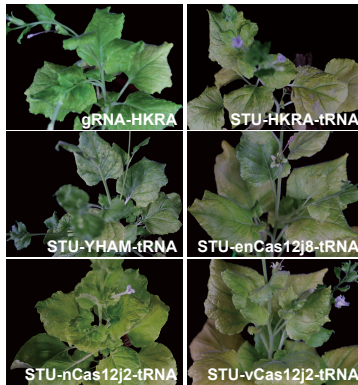

(c)

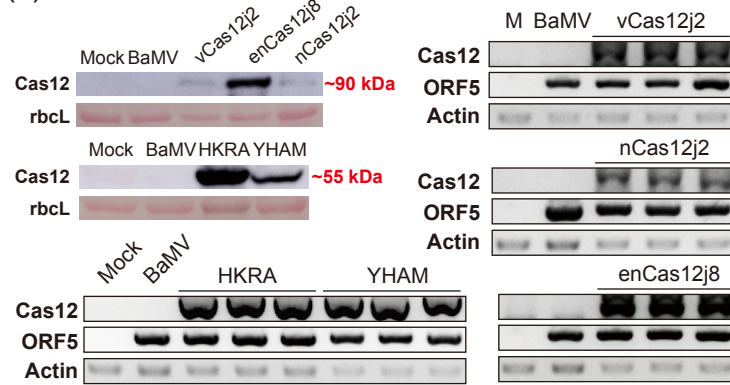

(d)

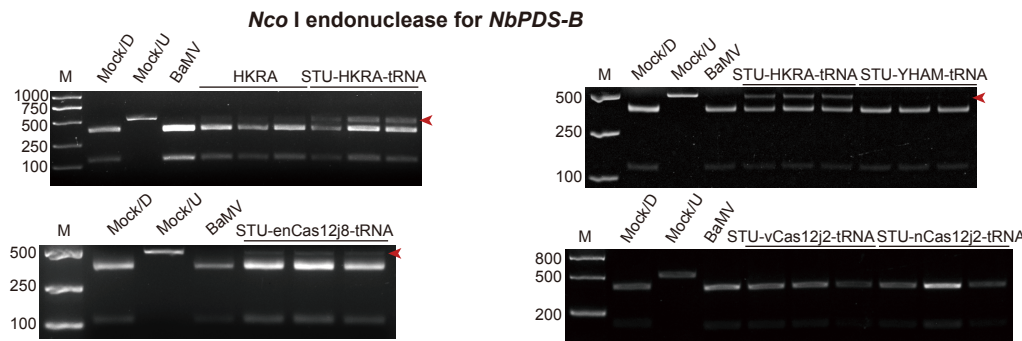

(e)

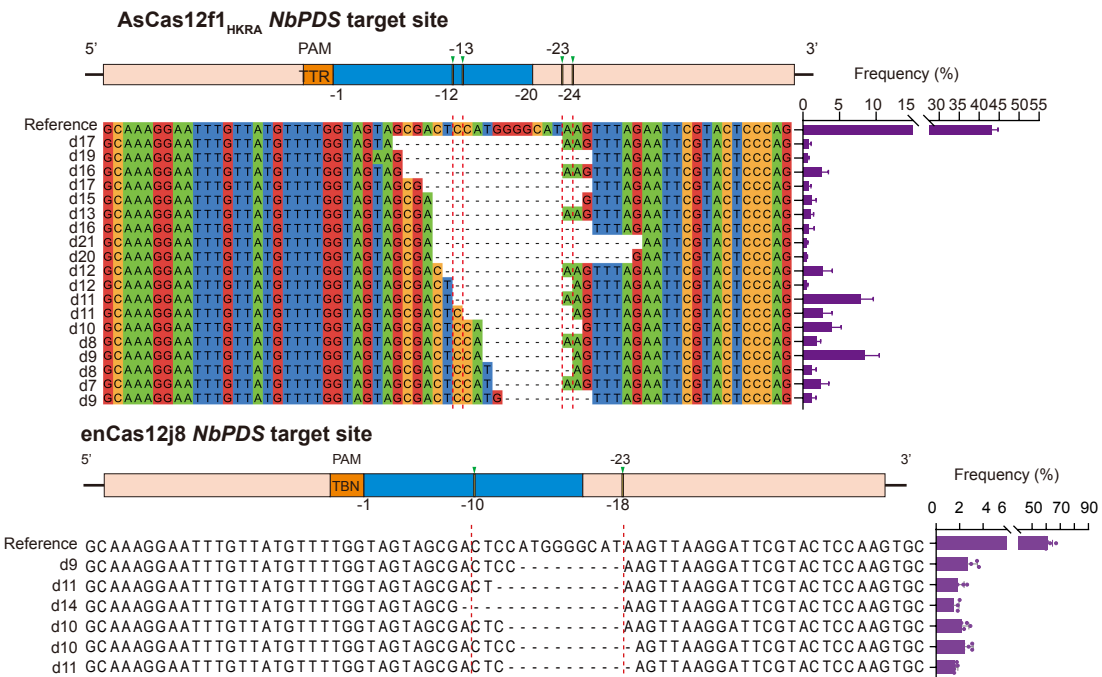

(f)

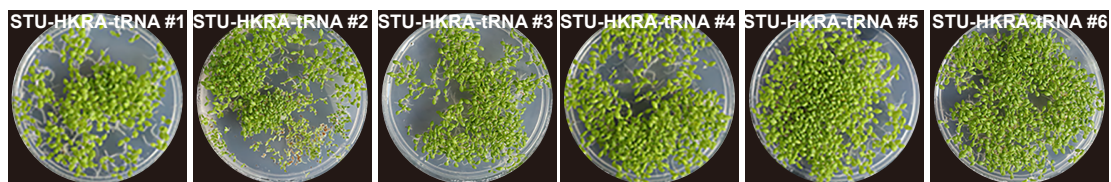

Figure S3

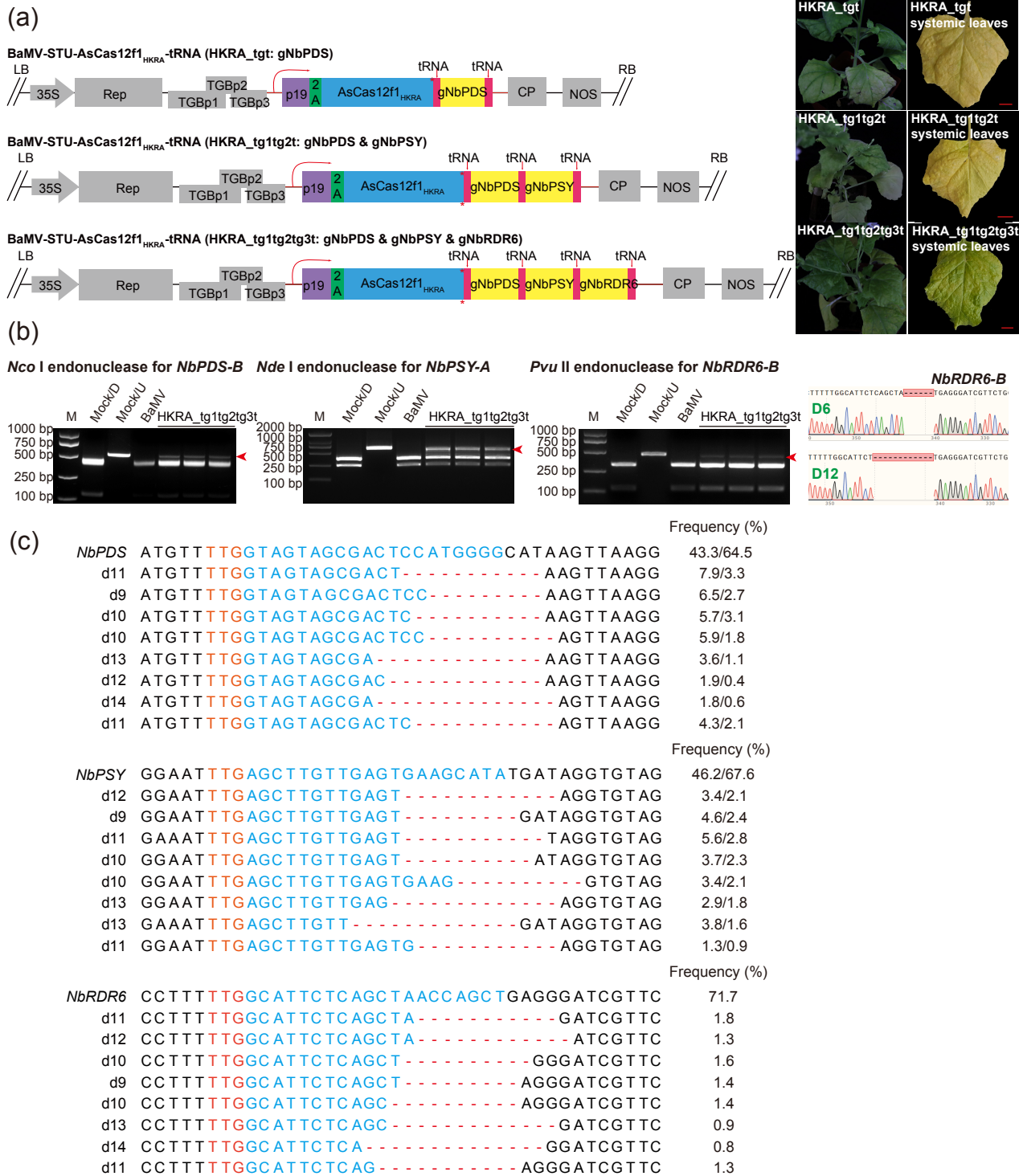

Figure S4

(a)

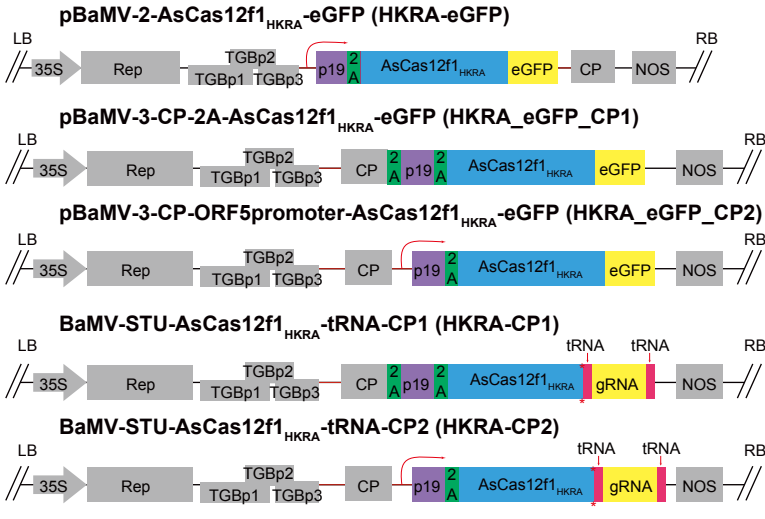

(c)

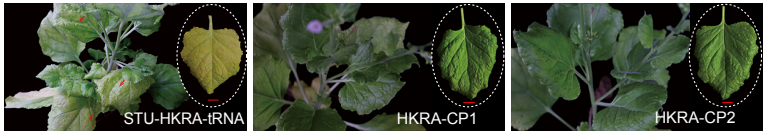

(b)

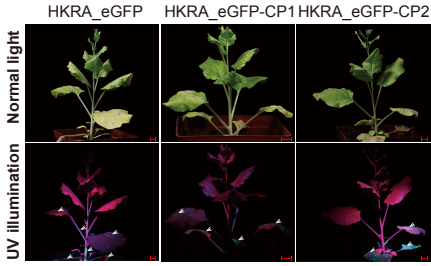

(d)

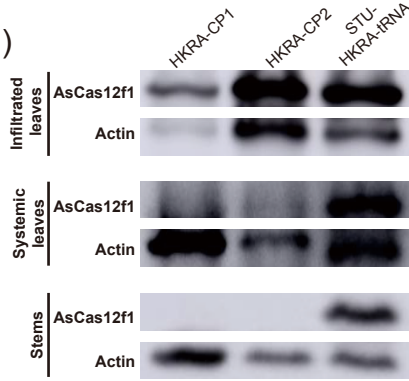

(e)

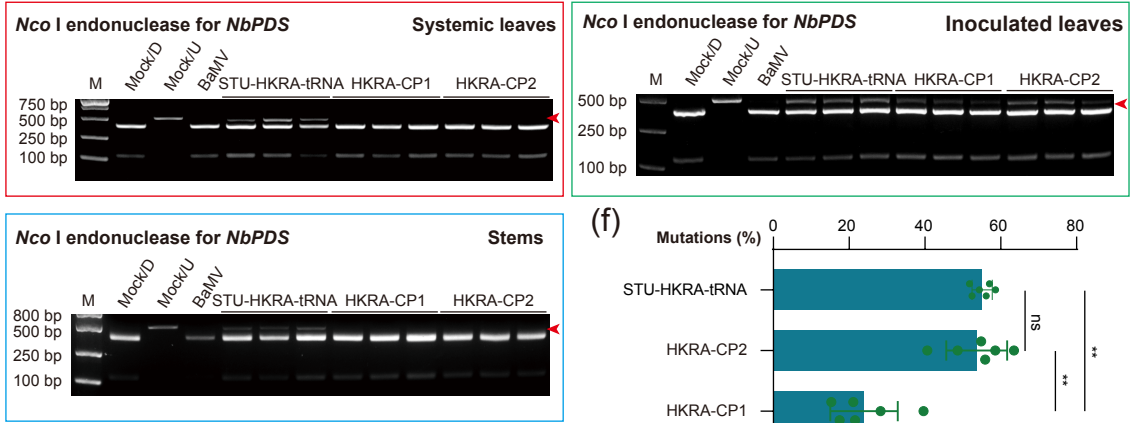

**Figure S5**

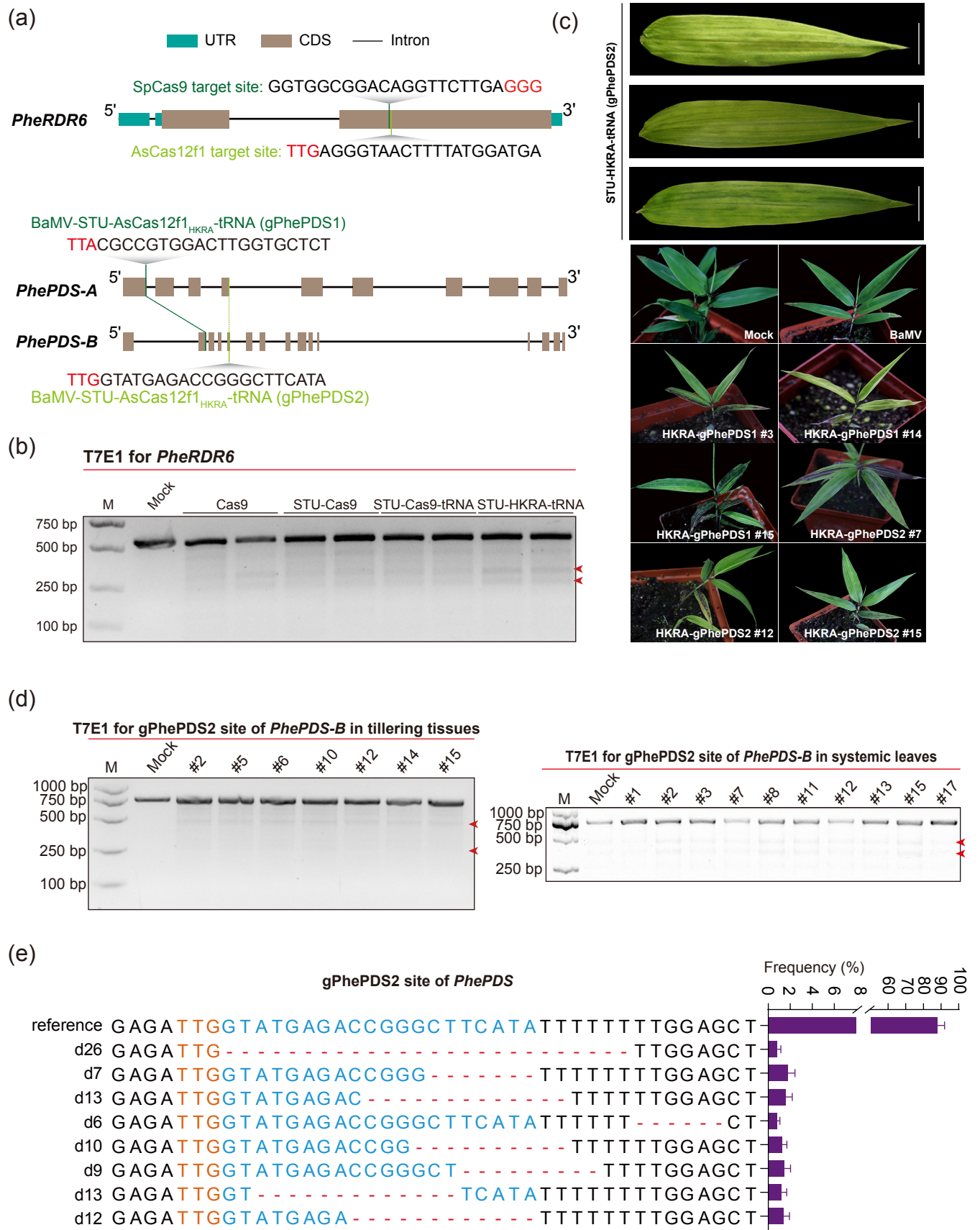

Supplement: Supplementary file 3 — Appendix S3: Figures S1–S5 (details provided in Appendix S3). Figure S1: BaMV‐mediated single transcript CRISPR‐Cas9 system. Figure S2: BaMV‐mediated single transcript CRISPR‐Cas12 system. Figure S3: Multiplexed endogenous genome editing. Figure S4: Evaluation of AsCas12f1HKRA‐tgtRNA insertions at distinct sites. Figure S5: Genome editing in Phyllostachys edulis. [file PBI-24-2220-s003.zip › pbi70474-sup-0003-AppendixS3/Supplementary Material Figures-S1-5.pdf]
